# Matrix Integrative Analysis (MIA) of Multiple Genomic Data for Modular Patterns

**DOI:** 10.3389/fgene.2018.00194

**Published:** 2018-05-29

**Authors:** Jinyu Chen, Shihua Zhang

**Affiliations:** ^1^NCMIS, CEMS, RCSDS, Academy of Mathematics and Systems Science, CAS, Beijing, China; ^2^School of Mathematical Sciences, University of Chinese Academy of Sciences, Beijing, China; ^3^Center for Excellence in Animal Evolution and Genetics, Chinese Academy of Sciences, Kunming, China

**Keywords:** bioinformatics, multi-dimensional genomics, matrix integrative analysis, data integration, module discovery, non-negative matrix factorization (NMF), partial least squares (PLS)

## Abstract

The increasing availability of high-throughput biological data, especially multi-dimensional genomic data across the same samples, has created an urgent need for modular and integrative analysis tools that can reveal the relationships among different layers of cellular activities. To this end, we present a MATLAB package, Matrix Integration Analysis (MIA), implementing and extending four published methods, designed based on two classical techniques, non-negative matrix factorization (NMF), and partial least squares (PLS). This package can integrate diverse types of genomic data (e.g., copy number variation, DNA methylation, gene expression, microRNA expression profiles, and/or gene network data) to identify the underlying modular patterns by each method. Particularly, we demonstrate the differences between these two classes of methods, which give users some suggestions about how to select a suitable method in the MIA package. MIA is a flexible tool which could handle a wide range of biological problems and data types. Besides, we also provide an executable version for users without a MATLAB license.

## Introduction

Biological systems are very complex, consisting of diverse components interacting with each other cooperatively. A key problem of biology is to investigate the relationships among different layers of cellular activity and thereby gain deep understanding of the underlying regulatory mechanisms. Meanwhile, the rapid development of high-throughput genomics technologies has accelerated the generation of large-scale genomic data at multiple levels simultaneously on the same samples. For example, The Cancer Genome Atlas (TCGA) project (The Cancer Genome Atlas Research Network, 2008) provides copy number variation, DNA methylation, microRNA and gene expression profiles for the same set of tumor samples. Thus, it is an essential and valuable task to develop tools that can explore combinatorial relationships among multiple layers of cellular activities. To this end, we present a MATLAB package, Matrix Integration Analysis (MIA), which implements four methods for modular and integrative analysis. The four methods are extensions of two classical techniques—non-negative matrix factorization (NMF) and partial least squares (PLS).

In this paper, we firstly provide a brief review about these two classes of methods and their applications in bioinformatics as well as four methods in the MIA package. We then focus on the details about MIA including its implementation, input data structures, and outputs. We also give a very detailed user manual about this package in our online guide file. Moreover, we summarize the characteristics of each method in the MIA package when applying to real data matrices, which provides users some practical suggestions about method selection. Particularly, in order to demonstrate the differences between these two classes of methods clearly, we compare NMF-based methods with PLS-based methods by applying them to some sets of simulated data matrices.

## Materials and equipment

### Brief review of NMF

The NMF technique, as an unsupervised and part-based learning method, has been increasingly applied to diverse fields including bioinformatics [e.g., high-dimensional genomic data analysis (Brunet et al., 2004; Kim and Park, 2007; Devarajan, 2008; Zhang et al., 2011, 2012; Li et al., 2012; Chen and Zhang, 2016)]. It decomposes a non-negative matrix X of size m × n into two non-negative matrices—a basis matrix W ∈ ℝ^m×k^ and a coefficient matrix H ∈ ℝ^k×n^, such that X ≈ WH, where k < min {m, n} (Paatero and Tapper, 1994; Lee and Seung, 1999). That is, data X is explained as a positive linear combination of basis vectors (the columns of W). To find an appropriate decomposition for matrix X, a number of cost functions are proposed (Paatero and Tapper, 1994; Lee and Seung, 1999, 2000; Cichocki et al., 2006, 2008; Dhillon and Sra, 2006; Fevotte et al., 2009). Two types of cost functions are often used to measure the distance between the original data matrix X and the reconstructed matrix WH. One is based on the Euclidean distance, that is, ${\text{min}}_{\text{W,H}}\|\text{X}-\text{WH}{\|}_{\text{F}}^{2}$ (Paatero and Tapper, 1994; Lee and Seung, 2000); the other is based on Kullback-Leibler (KL) divergence, that is, $\min_{\text{W,H}}\sum_{\text{i,j}}\left({\text{X}}_{\text{ij}}\log\frac{{\text{X}}_{\text{ij}}}{{\left(\text{WH}\right)}_{\text{ij}}}-{\text{X}}_{\text{ij}}+{\left(\text{WH}\right)}_{\text{ij}}\right)$, where X_ij_ represents the element of matrix X (Lee and Seung, 1999, 2000). Factored matrices W and H could be used to identify the underlying substructures in data matrix X.

The simplicity and efficiency of NMF make it have wide applications. For example, Brunet et al. (2004) and Moffitt et al. (2015) applied NMF to gene expression data to discover cancer subtypes. Compared to other traditional clustering methods, such as hierarchical clustering and k-means, NMF is able to capture local structures and provides a robust clustering of samples. Besides, NMF is able to identify genomic modules involved in similar biological functions using cancer genomic data, such as gene modules (Kim and Tidor, 2003) and mutation signature modules (Nik-Zainal et al., 2012; Alexandrov et al., 2013a,b; Kasar et al., 2015), which help us understand the underlying pathogenic mechanism of different types of cancers.

Several variants of NMF have been proposed by adding distinct constraints. For example, Feng et al. (2002) imposed sparseness constraints to matrix H and locality constraints to matrix W on the basis of KL divergence-type objective function, that is, $\min_{\text{W,H}}\sum_{\text{i,j}}\left({\text{X}}_{\text{ij}}\log\frac{{\text{X}}_{\text{ij}}}{{\left(\text{WH}\right)}_{\text{ij}}}-{\text{X}}_{\text{ij}}+{\left(\text{WH}\right)}_{\text{ij}}\right)+$ $\alpha \underset{i,j}{\sum }{\left(W^{T}W\right)}_{ij}-\beta$ $\underset{\text{i}}{\sum }{\left(\text{H}{\text{H}}^{\text{T}}\right)}_{\text{ii}}$ to learn spatially localized parts-based representation of visual patterns. In order to achieve feature selection, it is useful to explicitly control the sparse degree of W and H by imposing sparsity constraints (e.g., L_0_-, L_1_-, or L_2_-norm penalty) to W and H (Kim and Park, 2007; Peharz and Pernkopf, 2012). These sparse NMF methods not only perform the reconstruction of original data X, but also extract highly localized patterns. Orthogonal constraints are also commonly used in NMF (Ding et al., 2006; Wang et al., 2008; Strazar et al., 2016), that is, W^T^W = I and (or) H^T^H = I. Orthogonal NMF leads to rigorous clustering interpretation. Recently, Strazar et al. (2016) applied an orthogonal joint NMF framework to integrate multiple data for deciphering RNA binding patterns and predictions.

Network-regularized NMF (Cai et al., 2008; Wang et al., 2008; Zhang et al., 2011) incorporates prior knowledge such as interaction networks between features in data X into NMF framework such that linked features in the networks are sufficiently close to each other in the new representation space. For example, Zhang et al. (2011) utilized the same skill to add graph constraints about gene-gene and gene-microRNA interaction networks to NMF framework, and identified gene-microRNA co-modules which function cooperatively in the biological system.

Moreover, in order to simultaneously integrate more than one type of genomic data across the same set of samples, Zhang et al. (2012) proposed a joint NMF (jNMF) technique, which simultaneously decomposes multiple genomic data matrices X_i_ into a common basis matrix W and individual metagene matrix H_i_. Based on H_i_, they identified a number of multi-dimensional modules, each of which comprises a set of genes, microRNAs and methylation markers. Ray and Bandyopadhyay (2016) proposed a NMF based approach to integrate multiple biological networks, including gene co-expression network, PPI network and GO semantic similarity network, to predict the interactions between HIV-1 and human proteins.

Besides two-factor NMF, three-factor NMF is also an important class of matrix factorization techniques (Ding et al., 2006; Wang et al., 2008; Pei et al., 2015; Zitnik and Zupan, 2015; Zitnik et al., 2015), that is, X ≈ FSG. Such format provides a framework to perform biclustering of the rows and columns of matrix X by matrices F and G, respectively. Factored matrix S not only provides an additional degree of freedom making the approximation tightly, but also could indicate the relations between identified clusters. Recently, Zitnik and Zupan (2015) proposed a data fusion model by matrix factorization (DFMF), which can integrate multiple relationships between multiple object types as well as constraints for these object types. They applied it to fuse 11 data matrices including gene expression profiles, gene annotations with GO terms, and KEGG pathways and so on, to predict gene functions.

For the convenience of users, NMF including its variants has been implemented in several standard programming languages. For example, Gaujoux and Seoighe (2010) provided an R package implementing several NMF optimization algorithms and Qi et al. (2009) developed a NMF analysis plug-in in BRB-ArrayTools implemented in R for microarray gene expression analysis. There also exist a number of MATLAB packages for NMF, such as the package developed by Brunet et al. (2004) for metagenes and molecular pattern discovery; the NMF Toolbox developed by Li and Ngom (2013), which contains a variety of techniques for biological data mining; bioNMF proposed by Pascual-Montano et al. (2006), which is a standalone tool for classical NMF and contains a user-friendly graphical interface to demonstrate data analysis results.

For NMF, the number of latent factors k in the decomposition is a key parameter which needs to be pre-determined. For the application that using NMF to perform clustering, people often determine k based on a consensus clustering matrix by means of cophenetic correlation coefficient (Brunet et al., 2004; Zitnik and Zupan, 2015) and the stability of clustering. In addition, the distance between the original matrix X and the reconstructed matrix WH can also be used as a measure to select k (Kim and Tidor, 2003; Zitnik and Zupan, 2015; Zitnik et al., 2015). Recently, Wu et al. (2016) selected k when the learned basis matrix W achieves the lowest instability under different initial starting points in NMF algorithm. Particularly, in biological analysis, we could select k based on prior knowledge about the input data matrix X or functional enrichment analysis.

### Brief review of PLS

PLS is a multivariate regression method used to find the relations between an input matrix $\text{X}\in {\text{ℝ}}^{\text{m}\times {\text{n}}_{1}}$ and a response matrix $\text{Y}\in {\text{ℝ}}^{\text{m}\times {\text{n}}_{2}}$, both of which have the same rows (samples) (Wold et al., 2001; Rosipal and Kramer, 2006; Lê Cao et al., 2008; Chun and Keles, 2010; Li et al., 2012; Chen and Zhang, 2016). Compared to the classical linear regression, it works well for the data with small sample size and a large number of variables (m < n_1_). PLS decomposes matrices X and Y both with zero-mean variables into the form: X = TP^T^ + E, Y = UQ^T^ + F, where T, U are m × k matrices of k latent components describing the original data matrices in a low-dimensional space, and T, U can be constructed as a linear transformation of X, Y, respectively (that is, T = XW; U = YC); P, Q represent loading matrices to measure the relationships between the original variables and latent ones; E, F are residual matrices. PLS aims to maximize the covariance between X and Y by means of latent components T and U. One common type objective function of PLS is $\underset{\text{w},\text{ c}}{\max}\;{{\left[\text{cov}\;(\text{t},\text{ u})\right]}^{2}=\left[\text{cov}\;(\text{Xw},\text{Yc})\right]}^{2}$, subject to w^T^w = c^T^c = 1. Here, w and c are respectively one column of weight matrices W and C; similarly, t and u are respectively one column of latent component matrices T and U. If the columns of X and Y are zero-mean, cov(Xw, Yc) could be calculated by w^T^X^T^Yc/m. Since [cov (Xw, Yc)]^2^ = var (Xw) [corr (Xw, Yc)]^2^ var(Yc), thus latent variables t and u identified by PLS simultaneously take into account the requirements of maximal correlation between X and Y like Canonical Correlation Analysis (CCA), explaining as much variance as possible in both X- and Y-space like Principal Components Analysis (PCA). This model can be solved by the non-linear iterative partial least squares (NIPALS) algorithm (Wold, 1975; Wold et al., 2001; Rosipal and Kramer, 2006). Note that it only extracts one latent component t and u in one round of calculation. Loading vectors p and q are computed as the regression coefficients of X on t and Y on u, respectively: p = X^T^t/(t^T^t) and q = Y^T^u/(u^T^u).

Before calculating the next pair of latent variables t and u, matrices X and Y need to be deflated by subtracting their rank-one approximations. Different models have used different deflation forms (Rosipal and Kramer, 2006). If people want to model the symmetric relation between the two blocks X and Y, X and Y are deflated as X = X − tp^T^, Y = Y − uq^T^; If a regression model is needed, the computed latent components (column vectors in matrix T) are treated as good predictors of Y. Assume that a linear relation between t and u exists, U = TD + H, where D is a diagonal matrix and H is a residual matrix, thus X and Y are updated as X = X − tp^T^, Y = Y − tt^T^Y/(t^T^t).

PLS has been widely applied to various problems in bioinformatics (Boulesteix and Strimmer, 2005; Singh et al., 2016). For example, Boulesteix and Strimmer (2005) utilized PLS regression method to predict transcription factor activities by combining mRNA expression (Y) and DNA-protein binding data (X). Besides, in order to perform feature selection for high dimensional data, sparse PLS (SPLS) technique has been developed (Lê Cao et al., 2008; Chun and Keles, 2010), which imposes sparsity penalties to weight vectors w and c. For example, Lê Cao et al. (2008) used Lasso penalty to select biologically interpretable genes. Morine et al. (2011) used SPLS regression to assess relationships between dietary components and gene expression levels. Huang et al. (2004) also proposed a sparse PLS named Penalized PLS (PPLS) method, in which they selected key features by means of soft thresholding process to shrink the coefficients of some features to zero. Their results indicate the selected features by PPLS could provide more accurate prediction than traditional PLS and a random forest method. Furthermore, Liquet et al. (2016) proposed group PLS and sparse group PLS models, which take into account the group effects due to relationships among predictors (e.g., genes in the same pathway). However, they only consider non-overlapping groups, which may need to improve due to the popularity of overlapping groups in practice.

In addition, PLS can be combined with classification methods to solve classification tasks. For examples, Nguyen and Rocke performed a two-group tumor classification task on the microarray gene expressions by combining PLS and discrimination analysis (Nguyen and Rocke, 2002b). Furthermore, they extended this method to solve the multi-class cancer classification problem (Nguyen and Rocke, 2002a). Moreover, a number of methods combing SPLS with different classification models such as linear discriminant analysis, support vector machine and random forest are developed (Gutkin et al., 2009; Chung and Keles, 2010; Lê Cao et al., 2011). They could construct more accurate and efficient classifiers with selected features.

PLS has been widely implemented. For example, the Statistics and Machine Learning Toolbox module plsregress in MATLAB carries out PLS regression. The pls package provided by Mevik and Wehrens implements several PLS algorithms in R (Mevik and Wehrens, 2007). The gpls package (Ding, 2016) is able to accomplish classification using generalized partial least squares for two-group and multi-group classification. Particularly, plsgenomics developed by Boulesteix et al. is a PLS-based genomic analysis R package (Boulesteix et al., 2015), mainly implementing PLS methods for classification with microarray data and prediction of transcription factor activities from combined ChIP-chip analysis. Lê Cao et al. developed an R package, integrOmics, implementing SPLS, which could simultaneously integrate two types of “omics” variables that are measured on the same samples and realize variables selection using lasso penalization (Lê Cao et al., 2009). Chung et al. developed the spls package to perform sparse PLS regression and classification in R (Chung et al., 2013).

PLS also needs to determine the number of latent components. Cross-validation (CV) strategy is usually used to solve it (Wold et al., 2001; Boulesteix and Strimmer, 2005). Besides, the robustness of a PLS algorithm is also an important issue. It is well-known that the two popular algorithms NIPALS and SIMPLS for solving PLS are very sensitive to outliers in the data (Cummins and Andrews, 1995; Gil and Romera, 1998; Hubert and Vanden Branden, 2003; Serneels et al., 2005). Several robust PLS algorithms have been proposed including using iteratively reweighting technique, that is, assigning outliers detected by some way with low weights (Cummins and Andrews, 1995; Serneels et al., 2005); and estimating a robust covariance matrix instead of the previous one used in PLS (Gil and Romera, 1998; Hubert and Vanden Branden, 2003).

As for NMF and PLS, they are both powerful methods to capture the inherent structures of data. The optimization problems corresponding to them are not convex, thus it is very hard to find a global optimal solution. Besides, the number of latent components k needs to be pre-determined for both NMF and PLS. However, there are several differences between them. Firstly, NMF aims at exploring local patterns in one data matrix X. The identified local patterns are some features sharing high signals across a same subset of samples. PLS emphasizes regression analysis exploring the relationships between two types of features in matrices X and Y. The highly correlated features in X and Y are selected. Secondly, the constraints for them are distinct. NMF requires input data are non-negative whereas PLS needs centered data across samples as input just like simple linear regression.

### MIA: a matlab package for matrix integrative analysis

**Figure 1:**
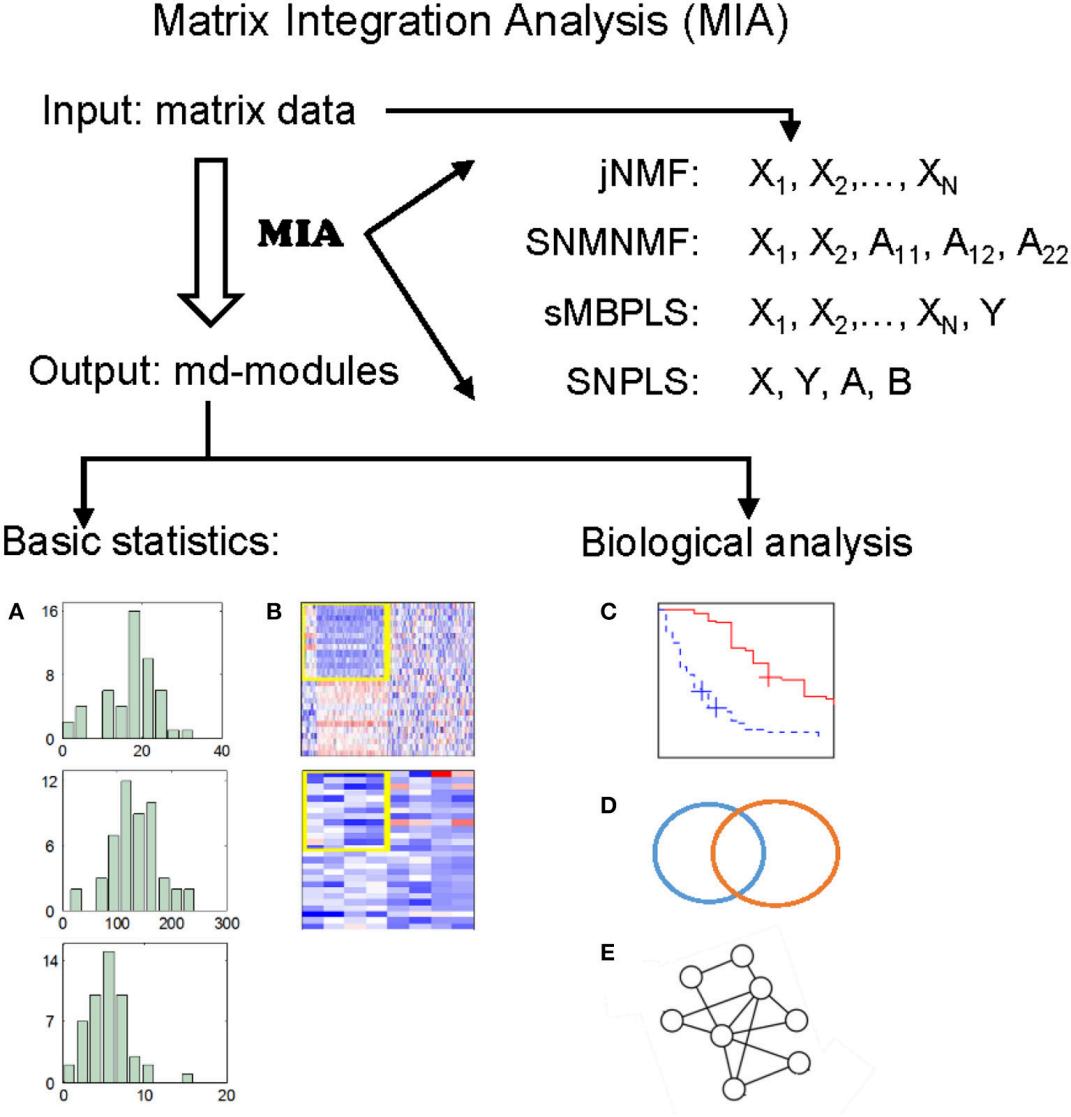
Illustration of MIA. MIA implements four methods with multiple matrices representing different biological features on the same samples (and prior network knowledge) as input, and discovers different types of multi-dimensional modules (md-modules) as output. MIA also provides several basic statistics about the md-modules, such as their sizes, member lists, size distributions **(A)**, and heatmaps **(B)**. The reported md-modules can be easily adapted for further biological analysis such as survival analysis **(C)**, various enrichment analysis **(D)**, and network analysis **(E)**.

The wide applications of NMF and PLS methods prove that NMF and PLS are two types of powerful tools for analyzing genomic data. Recently, we have developed four promising methods, by extending classical NMF and PLS, for detecting **m**ulti-**d**imensional modules (md-modules) in diverse genomic data (as well as prior network data) (Table 1): jNMF (**j**oint **NMF**) (Zhang et al., 2012), SNMNMF (**S**parse **N**etwork-regularized Multiple **NMF**) (Zhang et al., 2011), sMBPLS (**s**parse **M**ulti-**B**lock **PLS**) (Li et al., 2012), and SNPLS (**S**parse **N**etwork-regularized **PLS**) (Chen and Zhang, 2016). Currently, practical tools to integrate multi-dimensional genomic data are still lacking. To this end, we develop a unified **M**atrix **I**ntegration **A**nalysis (**MIA**) package, implementing these four methods as a set of MATLAB functions, to facilitate their adoption, promotion and evaluation (Figure 1).

**Table 1:** Brief summary of the four methods in MIA.

**Method**	**Description**	**Non-negative constraint**	**Network penalty**	**No. of matrices**	**References**
jNMF	Factorize multi-dimensional genomic data simultaneously to reveal multi-dimensional modules in an unsupervised manner.	Yes	No	Multiple	Zhang et al., 2012
SNMNMF	Incorporate prior networks into jNMF for two types of data to enhance co-module discovery.	Yes	Yes	Pairwise	Zhang et al., 2011
sMBPLS	Extend sparse PLS regression model for simultaneously analyzing multi-dimensional genomic data to reveal multi-dimensional regulatory modules.	No	No	Multiple	Li et al., 2012
SNPLS	Incorporate prior networks into sMBPLS for pairwise data matrices to reveal co-module patterns.	No	Yes	Pairwise	Chen and Zhang, 2016

### Brief review of the four methods in MIA

The four methods are developed for integrative and modular analysis for multiple genomic data as well as prior network data. We briefly review these methods and clarify some differences among them in terms of input data types, data formats, and module characteristics.

#### jNMF

jNMF enables users to simultaneously factor two- or multi-dimensional genomic data of the same set of samples. It discovers local patterns across the same subset of samples in multiple data matrices simultaneously. Note that matrices with negative elements must be transformed into non-negative matrices before analysis to satisfy the non-negative constraint. In this package, such matrices are transformed by X^*^ = [max (X, 0), max (−X, 0)], where each row represents one sample (users may adopt other ways to transform a matrix). More importantly, we extend the implementations in Zhang et al. (2012) to be suitable for any number of matrices, that is,

$$\begin{array}{l} \underset{\text{W},{\text{H}}_{\text{i}}}{\text{min}}\sum_{i\;=\;1}^{n}\|X_{i}\;-\;WH_{i}{\|}_{F}^{2},\text{s}.\text{t}.\text{ W}\geq 0,\;{\text{H}}_{i}\geq 0,\;i\;=\;1,\ldots ,\;n. \end{array}$$

#### SNMNMF

SNMNMF integrates prior networks relating to input variables into jNMF for pairwise case. For example, a gene-gene interaction network and a microRNA-gene interaction network have been adopted into this framework for discovering microRNA-gene co-modules (Zhang et al., 2011). By expressing networks as network-regularized penalties, SNMNMF makes the variables linked in these networks more likely to be placed into the same module, which will be biologically interpretable. Compared with the model in Zhang et al. (2011), SNMNMF implemented in this package can incorporate the links within one type of variables (${\text{A}}_{11}\in {\mathbb{R}}^{n_{1}\times n_{1}},\;A_{22}\in {\mathbb{R}}^{n_{2}\times n_{2}}$) and between the two types of variables (${\text{A}}_{12}\in {\mathbb{R}}^{n_{1}\times n_{2}}$) in a more flexible manner, where A_11_, A_22_, A_12_ are adjacency matrices for the three networks, that is,

$$\begin{array}{l} \underset{\text{W},{\text{H}}_{\text{i}}}{\text{min}}{\sum^{\text{​}}}_{i\;=\;1}^{2}{\left\|X_{i}-WH_{i}\right\|}_{F}^{2}-{\sum^{\text{​}}}_{1\leq i\leq j\leq 2}{\text{λ}}_{ij}Tr\left(H_{i}A_{ij}H_{j}^{T}\right)\;\\ \;+\gamma_{1}{\left\|W\right\|}_{F}^{2}+\;\gamma_{2}(\sum_{i}\|h_{i}^{\left(1\right)}{\|}_{1}^{2}+\sum_{j}\|h_{j}^{\left(2\right)}{\|}_{1}^{2}),\\ \text{ s.t. W}\geq 0,\;{\text{H}}_{i}\geq 0,\;i=1,2. \end{array}$$

where ${\text{h}}_{\text{j}}^{\left(1\right)}$ and ${\text{h}}_{\text{j}}^{\left(2\right)}$ denote the i-th and j-th columns of H_1_ and H_2_ respectively; λ_ij_, γ_1_, and γ_2_ are parameters respectively controlling the degree of network constraints, the growth of W and the sparsity of H_i_, which all need to be pre-defined by users according to their input data. The term in this objective function, $\text{Tr}\left({\text{H}}_{\text{i}}{\text{A}}_{\text{ij}}{\text{H}}_{\text{j}}^{\text{T}}\right)=\underset{\text{p},\text{q}}{\sum }{\text{a}}_{\text{pq}}{\text{h}}_{\text{p}}^{\left(\text{i}\right)\text{T}}{\text{h}}_{\text{q}}^{\left(\text{j}\right)}$, enforces “must-link” constraint such that features with known interactions in A_ij_ have similar coefficient profiles in matrix H_i_ and H_j_. We note that users can choose the networks they prefer to use in this framework by setting the corresponding parameters non-zero.

#### sMBPLS

sMBPLS extends standard PLS to discover associations between multiple input matrices and a response matrix in a sparse manner (Li et al., 2012). This method in the MIA package is implemented to explore the regression relationships between any number of input matrices and one response matrix, that is,

$$\begin{array}{l} \underset{W_{i},qb_{i}}{\text{max}}\;cov\left(t,u\right)-\sum_{i\;=\;1}^{n}\lambda_{i}\|w_{i}{\|}_{1}-\mu \|q{\|}_{1}\\ \text{with }t_{i}=X_{i}w_{i},\;u=Yq,\text{ and }t=\sum_{i\;=\;1}^{n}b_{i}t_{i}\\ \text{s}.\text{t}.\|w_{i}{\|}_{2}=1,\;\|q{\|}_{2}=1,\;{\;\|b\|}_{2}=1,\;i=1,\ldots ,n. \end{array}$$

where λ_i_, μ control the degrees of sparsity of weight vectors w_i_, q. In MIA, users need to provide several candidate values for each parameter and the program is able to select a group of proper parameters for input data by means of a cross-validation procedure.

#### SNPLS

SNPLS is designed for one input matrix ($\text{X}\in {\text{ℝ}}^{\text{m}\times {\text{n}}_{1}}$) and one response matrix ($\text{Y}\in {\text{ℝ}}^{\text{m}\times {\text{n}}_{2}}$). It adopts network-regularized constraints via the adjacency matrices ${\text{A}}_{11}\in {\mathbb{R}}^{n_{1}\times n_{1}}$ of a given network G_1_ for X and/or ${\text{A}}_{22}\in {\mathbb{R}}^{n_{2}\times n_{2}}$ of another network G_2_ for Y. This method in MIA enables users to make use of the network structures about input data X and response data Y if available flexibly, that is,

$$\begin{array}{l} \underset{W,q}{max}\;cov\;\left(t,u\right)-\gamma_{1}w^{T}L_{1}w-\gamma_{2}q^{T}L_{2}q-\lambda \|w{\|}_{1}-\mu \|q{\|}_{1}\\ \text{with }t=Xw,\;u=Yq.\\ \text{s}.\text{t}.\|w{\|}_{2}=1,\;\|q{\|}_{2}=1. \end{array}$$

where ${\text{L}}_{\text{i}}={\text{D}}_{\text{i}}^{-\frac{1}{2}}\left({\text{D}}_{\text{i}}-{\text{A}}_{\text{ii}}\right){\text{D}}_{\text{i}}^{-\frac{1}{2}},\text{ i}=1,2$. D_i_ is the degree matrix of graph G_i_, that is, the elements of D_i_: ${\text{d}}_{\text{kk}}=\underset{\text{j}}{\sum }{\text{a}}_{\text{jk}}$ and d_jk_ = 0, for j ≠ k. Similar with sMBPLS, the candidates of parameters γ_1_, γ_2_, λ, μ also need to be given, and the program will choose a proper group automatically.

## Stepwise procedures

### Implementation

To run MIA, all the input genomic data need to be combined into a single data matrix in which the rows correspond to samples and the columns correspond to genomic features. Different types of genomic data correspond to different sets of columns in the input matrix. Prior biological networks can also be given as inputs of SNMNMF and SNPLS. They are represented as network-regularized constraints for enhancing module discovery. One can run the main function of MIA with a pre-selected method to automatically perform computations on the input data matrix. The outputs include a set of text files and figures to describe the discovered md-modules (Supplementary Materials and Guide).

The installation of the MIA package is as follows:

1. Download the MIA package in the website: http://page.amss.ac.cn/shihua.zhang/software.html.
2. Unzip the package into a specific directory (e.g., ‘*D:/’*), and set the work path of MATLAB (e.g., ‘*D:/MIA/’*).
3. Load the data. In the MIA package, there is a folder named ‘*InputData*’ storing the demo input data for the four methods. For example, we load the data named ‘*InputDataForSNMNMF.mat’* as input of *SNMNMF*.
  >> *load(‘InputData/InputDataForSNMNMF.mat,”Input’);*
4. Run the main function *MIA.m* with a desired method. For example, we select *SNMNMF* for analyzing the loaded data.
  >> *MIA(Input, ‘SNMNMF’);*

Then, MIA automatically performs all computations and saves all the results into the directory ‘*MIA/SNMNMF/SNMNMF_Results/.’*

Besides, we also provide an executable version of MIA that does not require a MATLAB license. Next, we will describe how to define its input data and what outputs include. Its detailed implementation can be found in Supplementary Materials.

### Input data

MIA implements all four methods using the same structure variable to describe input data. This variable, named *Input*, includes the following components (Supplementary Materials):

*Input.data*: A matrix storing all the multi-dimensional data sequentially (e.g., *Input.data* = [X_1_, …, X_N_]). Each row corresponds to a specific sample and each column to a feature. The set of column indexes for each type of genomic data are recorded in *Input.XBlockInd*.

*Input.XBlockInd*: A matrix of size N × 2. The two elements in the i-th row give the start and end column indexes in *Input.data* for the i-th matrix – X_i_ (i = 1, …, N).

*Input.YBlockInd*: Its format is similar to *Input.XblockInd*, storing the column indexes of response matrix Y in *Input.data* for sMBPLS and SNPLS methods.

*Input.netAdj*: A symmetric adjacency matrix of a given network used for SNMNMF and SNPLS, where the features have the same order as in *Input.data*. This network combines the interactions between and within the variables in multiple types of data matrices. For example, for SNMNMF, *Input.netAdj* $=\left[{\text{A}}_{11},\;{\text{A}}_{12};\;{\text{A}}_{12}^{\text{T}},\;{\text{A}}_{22}\right]$, where A_11_, A_22_ are respectively adjacency matrices for the interaction networks about features in data matrices X_1_, X_2_; A_12_ is for the interaction network between the two types of features. The element of this matrix equals to 1 for linked features in the network, and 0 otherwise.

*Input.SampleLabel*: A vector recording sample labels.

*Input.FeatureLabel*: A vector recording feature names in *Input.data*. The i-th label corresponds to the i-th feature in *Input.data*.

*Input.FeatureType*: A vector recording the feature types in *Input.data*. For example, *Input.FeatureType*={‘Gene expression,’ ‘microRNA expression,’ ‘DNA methylation’}.

*Input.params*: A structure variable, storing all the parameters used in a specific method (e.g., the pre-defined number of md-modules, the parameters in its objective function).

For these four methods, there are three common parameters, including

- *Input.params.NCluster*: The pre-defined number of md-modules. For example, we may set *Input.params.NCluster* = 50.
- *Input.params.maxiter*: The maximal number of iterations in the algorithm. For example, we may set *Input.params*.*maxiter* = 100.
- *Input.params.tol*: The precision for convergence of the algorithm. For example, we may set *Input.params.tol* = 10^−6^.

Besides, each method has its own specific parameters. Here, we take SNMNMF as an example. Others can be found in Supplementary Materials.

For SNMNMF, its specific parameters contain:

- *Input.params.nloop*: The number of repeating times to run this algorithm. To obtain a robust and good solution, this algorithm is run for multiple times repeatedly, and the solution with the minimal value of objective function is accepted. For example, we may set *Input.params.nloop* = 10.
- *Input.params.thrd_module*: A non-negative vector of size 1 × 3 to select features in md-modules. *Input.params.thrd_module*(i+1) is the threshold for selecting the i-th type of features in *Input.data* (i = 1,2). The first one is for selecting samples. The larger they are, the smaller number of features are selected. Users can set it based on the proper size of md-modules they think. For example, we may set *Input.params.thrd_module* = [1,1,1].
- *Input.params.thrNet11, Input.params.thrNet12, Input*.*params*.*thrNet22*: Three non-negative parameters are respectively set for the network constraints about network A_11_, A_12_, A_22_ in the objective function. User can choose networks they prefer to use in the framework by setting the corresponding parameters non-zero. For example, if *Input.params.thrNet11* = 0, network A_11_ will not be used.
- *Input.params.thrXr, Input.params.thrXc*: Two non-negative numbers are set for W-related and H_i_-related constraints respectively in the objective function. They control the degree of sparsity of matrices W and H_i_. For example, we may set *Input.params.thrXr* = 10, *Input.params.thrXc* = 10.

In addition, for the components that are not used in certain methods (e.g., *Input.YBlockInd* in jNMF and SNMNMF and *Input.netAdj* in jNMF and sMBPLS), users can set them null or just ignore them.

We note that MIA is able to partition *Input.data* into corresponding data matrices as input for each method automatically. We also provide a set of demo data in the folder named *InputData*. Here, we provide an example for constructing the input data used in SNMNMF. Suppose that one wants to identify 50 microRNA-gene co-modules by integrating gene expression profiles (${\text{X}}_{1}\in {\mathbb{R}}^{385\times 12456}$) and microRNA expression profiles (${\text{X}}_{2}\in {\mathbb{R}}^{385\times 559}$) across the same set of tumor samples, as well as gene interaction network G_1_ and gene-microRNA interaction network G_2_. Network G_1_ can be expressed by adjacency matrix A_11_ = (_*a*_*ij*_)12456 × 12456_, where a_ij_ = 1 if gene i and gene j are linked in network G_1_. Similarly, G_2_ is expressed by adjacency matrix ${\text{A}}_{12}\in {\mathbb{R}}^{12456\times 559}$. If microRNA interaction network is not available, the corresponding adjacency matrix A_22_ is defined as ${\text{A}}_{22}=0\in {\mathbb{R}}^{559\times 559}$.

For example, we could define input data *Input* as below and then save it as ‘*InputDataForSNMNMF.mat’* (Figure S1):

**Table:** 

*Input.data* = [X_1_, X_2_];
*Input.XBlockInd* = [1,12456;12457,13015];
Input.YBlockInd = [];
*Input.netAdj* = [${\text{A}}_{11},{\text{A}}_{12};{\text{A}}_{12}^{\text{T}},{\text{A}}_{22}$];
*Input.SampleLabel* = {‘TCGA*-*24*-*1105*-*01A′; …;
‘TCGA*-*13*-*0793*-*01A’};
*Input.FeatureLabel* = {‘SFRS8′; …;‘SCN3A’; ‘hsa*-*mir*-*488′;
…;‘hsa*-*mir*-*874’};
*Input.FeatureType* = {‘Gene’,‘miRNA’};
*Input.params.NCluster* = 50;
*Input.params.maxiter* = 100;
*Input.params.tol* = 10^−6^;
*Input.params.nloop* = 10;
*Input.params.thrd_module* = [1,1,1];
*Input.params.thrNet11* = 10^−4^; Input.params.thrNet12 = 0.01;
*Input.params.thrNet22* = 0;
*Input.params.thrXr* = 10; Input.params.thrXc = 10;

### Output results

Given input data and desired method, MIA automatically performs all computations and saves all the results in a specific folder named ^***^_Results, where ^***^ represents the pre-selected method name. Here, we continue taking SNMNMF for example (for other methods, please refer to Supplementary Materials). We load the data constructed above and type these commands in the command window of MATLAB:

>> *load(‘InputDataForSNMNMF.mat’,‘Input’)*
>> *MIA(Input, ‘SNMNMF’);*

**Figure 2:**
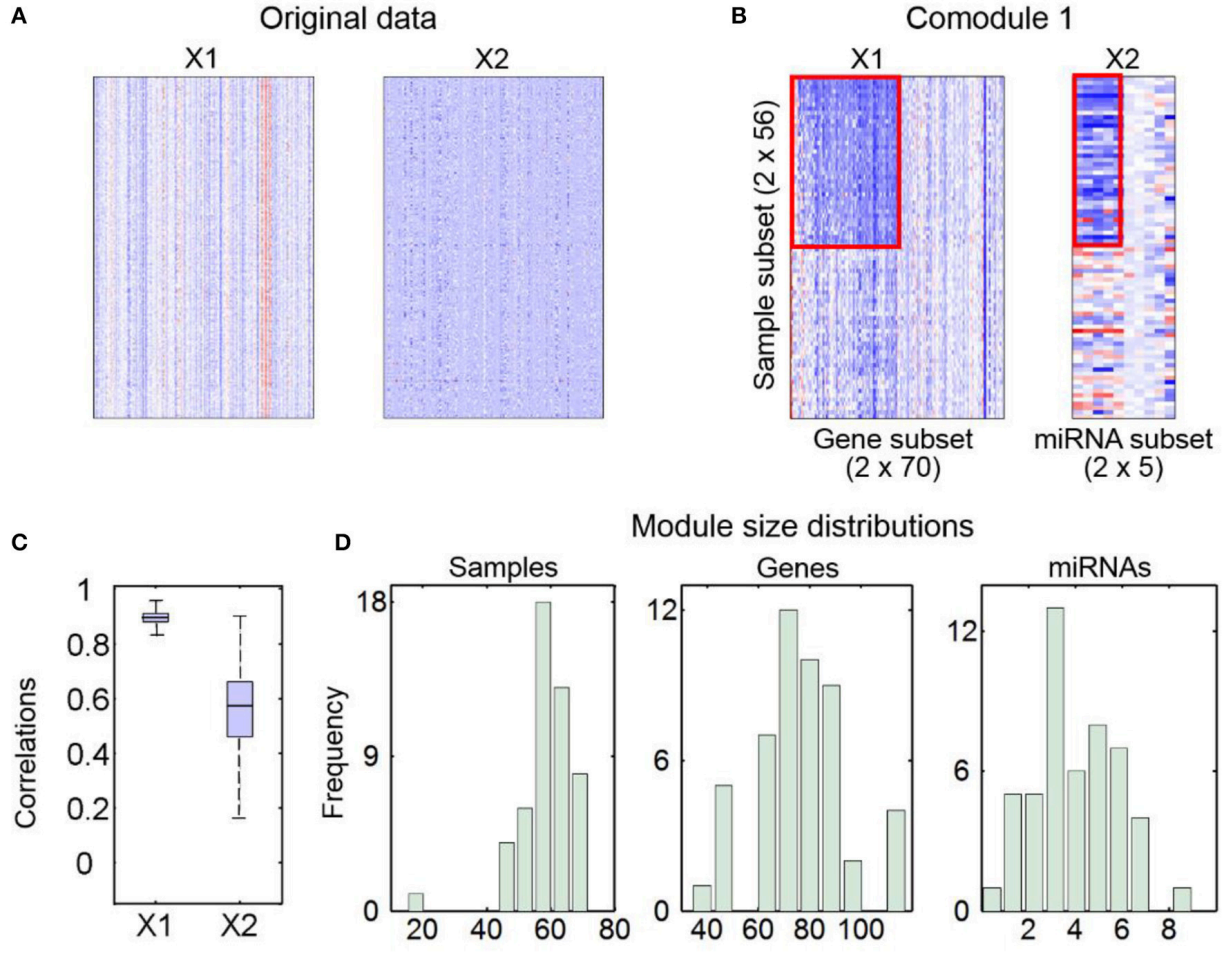
An example for output figures of SNMNMF. **(A)** Heatmaps of input data. **(B)** An example for heatmaps of identified md-modules (circled in red lines) and randomly selected features for comparison. **(C)** Sample-wise correlations between original data and corresponding reconstructed data by factorized matrices. **(D)** Size distributions for three types of components in md-modules.

All the results are saved in the directory ‘*MIA/SNMNMF/SNMNMF_Results/’*. Referring to the results, there are four parts (Figures S2–S4):

1. The first part is a MATLAB data file, named ‘*SNMNMF_Results.mat*’, saving key variables calculated by this pre-selected method.
2. The second part contains some figures, including heatmaps of input data (Figure 2A) and identified md-modules (Figure 2B), sample-wise correlations between input data and reconstructed data (Figure 2C) and size distributions for multi-type module members (Figure 2D).
3. The third part includes two text files, recording module members and objective function values during iterations.
4. The last part includes some folders, named as ‘^***^Lists’, where ^***^ represents the feature type defined by input variable ‘*Input.FeatureType*’ (e.g., “Gene,” “microRNA”). In each folder, there are a number of text files, each of which is a list of one type of components in one identified md-module.

Users can further analyze the biological significance of identified md-modules. For example, using the signals calculated by MIA, they can stratify patients into groups and then conduct Kaplan-Meier survival analysis to judge whether this module is related to clinical characteristics. They can also perform various functional enrichment analysis on the set of md-modules to gain insights into their functions. They may also conduct network analysis using tools like IPA, and/or construct a multi-level network for each md-module to explore the underlying relationships among features further.

## Results

### Biological applications

A number of biological analysis tools have been designed for one or two types of genomic data (Shen et al., 2009; Xu et al., 2015; Kowalski et al., 2016; Qin et al., 2016). Systematic analysis of multiple types of data for discovering biological relevant combinatorial patterns are currently limited. Here, the MIA package enables users to study the complex relationships and/or modular characteristics among multiple types of variables by integrating diverse types of large-scale omics data. For example, Zhang et al. applied jNMF to DNA methylation profiles, microRNA expression, and gene expression data from TCGA ovarian cancer dataset (Zhang et al., 2012). They identified a number of md-modules consisting of mRNA, microRNA, and methylation markers. These md-modules reveal multi-level vertical associations and cooperative functional effects. Besides, these md-modules can stratify patients into groups with distinct clinical characteristics.

Zhang et al. used SNMNMF to identify microRNA-gene regulatory co-modules by integrating gene and microRNA expression profiles as well as gene-gene and microRNA-gene target interaction networks (Zhang et al., 2011). The identified co-modules are enriched in known crucial functional sets, such as nuclear division, immune system process, and so on. Meanwhile, the co-modules are enriched with cancer-regulated genes and microRNA, suggesting the co-modules have strong implication in cancer. Furthermore, from the perspective of network analysis, these co-modules shed light on regulatory circuits.

Li et al. utilized sMBPLS to integrate four types of genomic data and identified multi-layer gene regulatory modules, including copy number variations, DNA methylation markers, microRNAs and genes, each of which constructs a local “gene expression factory” (Li et al., 2012). These modules reveal synergistic functions across multiple dimensions and facilitate regulatory analysis via using the modules to build multi-layer molecular interaction network.

Chen and Zhang applied SNPLS to gene expressions, drug response and gene network data to study (anti-)correlated gene-drug associations (Chen and Zhang, 2016). They identified a number of gene-drug co-modules, which show distinct biological relevance and significant drug-gene connections. These gene-drug co-modules reveal multiple-to-multiple relationships between drugs and their targets, and provide us insights into potential drug targets and drug combinations for cancer therapy.

In addition, MIA can have more applications. For example, it can discover relationships between species by integrating their gene expression profiles across multiple conditions. MIA can also predict transcription factor activities from combined analysis of microarray and ChIP-seq data. In summary, MIA is a flexible tool which can be applied to many problems involving diverse type of features of the same sets of samples.

These four methods of MIA can discover joint modular patterns when applied to distinct types of genomic data. They can be alternatively used in many situations. However, they also have different characteristics. Firstly, depending on whether users have network knowledge, users can select the methods with network constraints (SNMNMF and SNPLS) or without (jNMF and sMBPLS). Secondly, PLS-based methods emphasize regression analysis while NMF-based ones emphasize the identification of local patterns among data. Just like applications of jNMF (Zhang et al., 2012) and SNMNMF (Zhang et al., 2011), the integrative genomic data blocks are regarded equally. We can infer the identified genomic markers function cooperatively in biological system, but could not conclude that the dysregulation of some features leads to activity change of the others. Contrasting to NMF-based methods, PLS-based methods aim to describe the relationships between predictors in X and response variables in Y via regression analysis, expecting to predict how response variables change when predictors change, where X and Y are not treated symmetrically. For example, in Li et al. (2012), the identified modules by sMBPLS actually indicate the gene components are regulated by other components—copy number variations, DNA methylation markers and microRNAs, whose changes probably contribute to the changes of gene expression. Thus, before choosing method, we should have an assessment for relationships between data blocks. Thirdly, the constraints for these two classes of methods are distinct. NMF-based methods require non-negativity for input data matrices. For normalized genomic data, in some cases, they contain negative elements, and need to pre-process to satisfy the non-negativity constraint, which is unavoidable to change original data structure more or less, and leads to some effects on results. As for PLS-based methods, all the data blocks need to be centered across samples, which is a common way of normalization for genomic data.

We are eager to show the differences of NMF- and PLS-based methods in detail by applying them to real genomic data. However, it is hard to evaluate their performances, since the golden standards for real data are lacking. Thus, we employ an alternative way to show their difference based on simulated data.

### Simulation study

Here, we apply jNMF and sMBPLS to a set of simulated data matrices X and Y to demonstrate key differences between NMF- and PLS-based methods (Supplementary Materials). We believe that NMF-based methods tend to identify substructures with high absolute signals, whereas PLS-based ones prefer to discover patterns with strong correlations. Thus, we construct simulated data sets embedding two types of co-module structures—one type is module members with high absolute values across the same subset of samples; the other type is members with strong correlations. According to our assumption that these two types of methods have different preferences when detecting co-modules, two different types of golden standards for co-module members (G^(1)^ and G^(2)^) that jNMF and sMBPLS should identify respectively are created. We name G^(1)^ as NMF-based standard and G^(2)^ as PLS-based standard. If the co-modules identified by jNMF more approximate to G^(1)^ than those by sMBPLS and the co-modules by sMBPLS are more close to G^(2)^ than jNMF, it will verify the differences between these two types of methods as what we think. In the simulated data matrices X and Y, we embed five co-modules (Supplementary Materials), thus ${\text{G}}^{\left(\text{i}\right)}=\left\{{\text{G}}_{1}^{\left(\text{i}\right)},{\text{G}}_{2}^{\left(\text{i}\right)},\ldots ,{\text{G}}_{5}^{\left(\text{i}\right)}\right\},\text{ i}=1,2$, where ${\text{G}}_{\text{k}}^{\left(\text{i}\right)}$ is the member set for the k-th co-module in the NMF- or PLS-based standard. We use relevance score to measure the degree of similarity between the real co-modules G^(i)^ and the identified ones M = {M_1_, M_2_, …, M_5_} by jNMF or sMBPLS:

$$\begin{array}{l} \text{Relevance }\left(M,G^{\left(i\right)}\right)=\frac{1}{5}\sum_{M_{j}\in M}\underset{G_{k}^{\left(i\right)}\in G^{\left(i\right)}}{\max}s\left(M_{j},G_{k}^{\left(i\right)}\right),\\ \;s\left(M_{j},G_{k}^{\left(i\right)}\right)=\frac{\left|M_{j}\cap^{\text{​}}G_{k}^{\left(i\right)}\right|}{\left|M_{j}\cup^{\text{​}}G_{k}^{\left(i\right)}\right|}, \end{array}$$

where $\left|{\text{M}}_{\text{j}}\cap {\text{G}}_{\text{k}}^{\left(\text{i}\right)}\right|$ is the number of features in their intersection, and $\left|{\text{M}}_{\text{j}}\cup {\text{G}}_{\text{k}}^{\left(\text{i}\right)}\right|$ is the number of features in their union.

**Figure 3:**
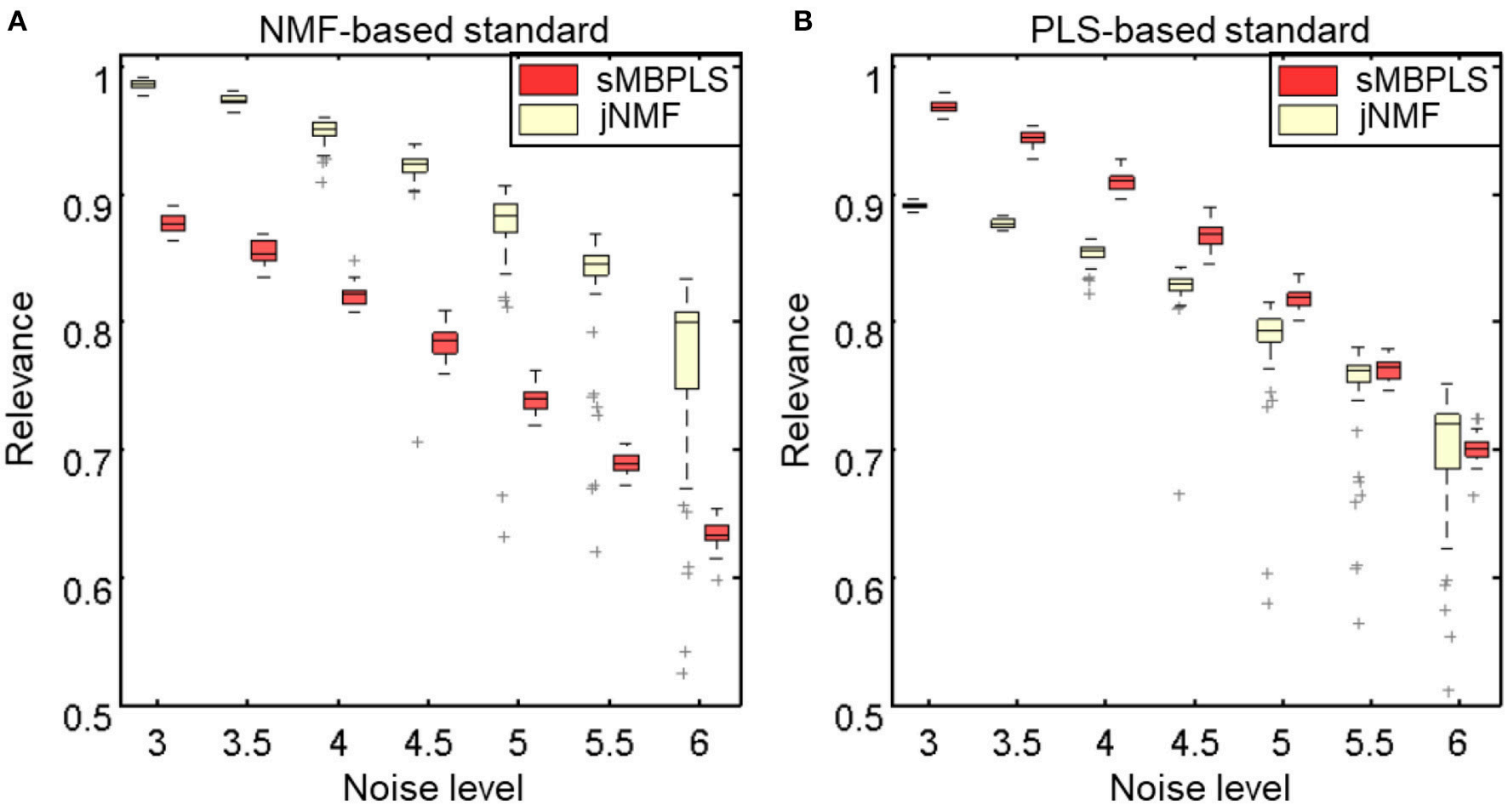
Comparison of jNMF and sMBPLS in terms of relevance scores under two types of golden standards, derived by the characteristics of NMF method **(A)** and PLS method **(B)**. Here, we apply jNMF and sMBPLS to 50 sets of simulated data matrices for each level of data noise, respectively.

We can clearly see that the relevance scores of jNMF are higher than sMBPLS under NMF-based standard (Figure 3A, Figures S7A–S11A), which shows the co-modules identified by jNMF are more similar to G^(1)^ than sMBPLS. Thus, jNMF is indeed able to discover some patterns that sMBPLS could not find well. For example, for the first embedded module in simulated data Y (i.e., features from the first to 100th column and from the 201st to 230th column), the first 100 components are highly positive or negative correlated with each other, but among them, the last 50 ones have rather low signals. The other 30 components (from the 201st to 230th column) have high signal with similar magnitude to the first 50 components, but they have weak correlations with the first 100 components. It shows jNMF identifies features from the first to 50th column and from the 201st to 230th column, whereas sMBPLS discovers those from the first to 100th column. On the other hand, sMBPLS performs better than jNMF under PLS-based standard (Figure 3B, Figures S7B–S11B). sMBPLS could identify highly correlated components, not only for samples but also for features in X and Y, although they may have slightly low signals, which jNMF often disregards. For example, the second co-module (i.e., samples from the 111st to 200th row and features from the 201st to 400th column of matrix X and those from the 101st to 200th column of matrix Y) has very low signal values across the first 40 samples in matrices X and Y, but these samples are closely negatively correlated with the other 50 samples. As a result, sMBPLS discovers all these 90 samples as components of the second co-module, while jNMF just identifies the last 50 samples.

Although the accuracy of identified co-modules by jNMF and sMBPLS both decreases with the data noises increasing, the relevance scores for sMBPLS decreases faster than jNMF, which may suggest that sMBPLS is more sensitive to data noise than jNMF. In particular, for the last co-module, the golden standards for both methods are the same, but jNMF performs better than sMBPLS under different levels of data noises (Figure S11). We guess that high correlation pattern is much easier to be affected compared to high signal one with the increase of data noise.

Besides, the scores for sMBPLS gradually approach to jNMF with data noise increasing (Figure 3B). Correlations between features of relatively high signals are more difficult to be weakened by the same level of data noise than those of low signals. Thus, with data noise increasing, jNMF and sMBPLS tend to find similar co-modules with large signals and high correlations.

## Conclusion and discussion

The advance of biological techniques makes multi-dimensional large-scale omics data available, which provides us opportunities to study the complex biological system from the perspective of multi-layer regulatory programs. Therefore, it is necessary to develop a tool to simultaneously integrate multi-dimensional biomedical data. Here, we present a MATLAB package, MIA, to conduct integrative and modular analysis for multi-dimensional genomic data across the same sets of samples as well as prior network knowledge to decode the relationships among different levels of cellular activities.

Although these four methods in the MIA package are all used to identify modular patterns in data matrices, users could select an appropriate one based on their own purpose and data characteristics. We have demonstrated the specificity of these two types of methods using simulation test, which also provides clues about the method applicability. For example, NMF-based methods tend to identify local patterns with high absolute signals, whereas PLS-based methods are essentially regression methods, thus they are likely to extract the components with strong correlations between predictors and response variables.

Since these problems are not convex, it is very hard to find a global optimal solution. For NMF-based methods, users could run the algorithm for several times repeatedly by setting the input parameter *Input.params.nloop* in MIA, and then the solution with the minimal value of objective function is used for further analysis. Thus, users could obtain a consistent solution by this strategy. When solving the optimization problems for PLS-based methods, the initial iteration point of algorithm have much effects on the solution. A good starting point helps the algorithm converge rapidly. In MIA, instead of generating a random starting point, we choose the solution of the original PLS problem without any constraints as an initial point to speed up the algorithm and meanwhile make its solution more stable.

MIA may run slowly when applying to multiple large-scale data sets. Thus, it is better to filter some features by data pre-processing to reduce the dimensions of input data. In the future, we will further optimize the programs in MIA to make it more efficient for large-scale data integrative analysis. In MIA, we respectively choose the most common used algorithms to solve them. We use multiplicative update rules for NMF-based methods, and non-linear iterative partial least squares (NIPLS) algorithm for PLS-based methods. We will provide users several other efficient optimization algorithms for choice. Besides, R language is widely used among researchers, thus we will prepare the version implemented in R code soon to facilitate its adoption.

In summary, MIA is very easy to use and does not require high-level programming skills. We expect it will become a routine exploratory tool for diverse biological problems.

## Availability

The MIA package is available at http://page.amss.ac.cn/shihua.zhang/software.html.

## Requirements

It works in MATLAB R2013a or later in Windows (64-bit). For users without a MALTAB license, MATLAB R2015b Runtime for Windows (64-bit) is required to install it.

## Author contributions

JC and SZ designed this study. JC and SZ performed the experiments and statistical analysis. JC and SZ interpreted the results and written the manuscript. All authors read and approved the final manuscript.

### Conflict of interest statement

The authors declare that the research was conducted in the absence of any commercial or financial relationships that could be construed as a potential conflict of interest.
