# Supplementary material for: Matrix Integrative Analysis (MIA) of Multiple Genomic Data for Modular Patterns
Source: Front Genet. 2018 May 29;9:194. doi: 10.3389/fgene.2018.00194 (PMC5992392; doi:10.3389/fgene.2018.00194)
Supplement: Supplementary file 1 [file Presentation_1.PDF]

Supplementary Materials for
"Matrix integrative analysis (MIA) of multiple genomic data for modular patterns"

1 Description

MIA (Matrix Integration Analysis) is a MATLAB package for modular and integrative analysis of multiple genomic data to decode the relationships among different levels of cellular activities. It works in MATLAB R2013a or later. Users are expected to have basic MATLAB knowledge. In addition, we also provide an implementation that does not require a MATLAB license.

This package implements and extends four methods including jNMF (**j**oint **NMF**) (Zhang *et al.*, 2012), SNMNMF (**S**parse **N**etwork-regularized **M**ultiple **NMF**) (Zhang *et al.*, 2011), sMBPLS (**s**parse **M**ulti-**B**lock **P**artial **L**east **S**quare) (Li *et al.*, 2012) and SNPLS (**S**parse **N**etwork-regularized **P**artial **L**east **S**quare) (Chen and Zhang, 2016). It can be used to identify **m**ulti-**d**imensional modules (md-modules) by integrating multi-dimensional genomic data such as copy number variation (CNV), DNA methylation, gene expression, microRNA expression profiles and/or molecule network.

Besides, we also provide a guide to describe the details about all the MATLAB functions used in MIA package. It includes a brief introduction to each method, the definitions and formats of input and output arguments, the usage of each function and so on.

2 Implementation

2.1 Users with a MATLAB license

Requirements: It works in MATLAB R2013a or later FOR Windows (64-bit).

1) Download MIA pacakge in the website: http://page.amss.ac.cn/shihua.zhang/software.html.

2) Unzip the package into a specific directory (e.g., *'D:/'*), and set the work path of MATLAB (e.g., *'D:/MIA/'*).

3) Load the data. In MIA package, there is a folder named *'InputData'* storing the demo input data for these four methods. For example, we load the data as input for SNMNMF.

   >> *load('InputData/InputDataForSNMNMF.mat','Input')*;

4) Run the main function *MIA.m* with a desired method. For example, we select SNMNMF for analyzing the loaded data.

   >> *MIA(Input, 'SNMNMF')*;

Then, MIA automatically performs all computations and saves all the results into the path *'MIA/SNMNMF/SNMNMF_Results/'*.

2.2 Users without a MATLAB license

Requirements: It works in Windows (64-bit) operating system. The Windows 64-bit version of MATLAB Runtime for R2015b is required to install which is available from the MathWorks Web site http://www.mathworks.com/products/compiler/mcr/index.html.

1) Prepare input data and store them in the path *'D:/MIA/InputData/'*. In this folder, we provide input data for each method as examples. For each method, there are two Excel files (one is input data matrices and the other one is input parameters), each of which includes several sheets. Users need to arrange their data in the same way as those example files in this folder we provide. Note that, each sheet is renamed as the corresponding variable name as described in our manuscript.

2) Open the Command Prompt (*cmd.exe*). Set the current path as where MIA package is located, e.g., *'D:/MIA'*.

3) Produce MATLAB data files (**.mat*) for selected method by running *PreInputData.exe*. Taking SNMNMF for example, type the command as below:

   PreInputData.exe *./InputData/DataForSNMNMF.xlsx* *./InputData/ParametersForSNMNMF.xlsx* *./InputData/InputDataForSNMNMF.mat* *SNMNMF*

   Note that, the first argument of *PreInputData.exe* is the name of Excel file storing input data matrices; the second one is the name of Excel file recording all the input parameters; the third one is the output file name given by users. Its output is a MATLAB-data file, saving all the input data for some method; the last one is the method name of what the produced input data is used for. The produced new data file (e.g., *'InputDataForSNMNMF.mat'*) is saved in the directory *'D:/MIA/InputData/'*.

4) Run *MIA.exe*. Type the command as below:

   MIA.exe *./InputData/InputDataForSNMNMF.mat* *SNMNMF*

   The first argument of *MIA.exe* is the input data file name and the second one is the selected method name. For each method, the results are saved in their own directory. For example, the results of running SNMNMF are saved in *'./MIA/SNMNMF/SNMNMF_Results/'*.

Next, we describe how to define input data and what output results include in detail.

3 Input data

To facilitate the usage, MIA package implements the four methods using variables with the same structure to describe input data. This variable, named *Input*, includes *Input.data*, *Input.XBlockInd*, *Input.YBlockInd*, *Input.netAdj*, *Input.SampleLabel*, *Input.FeatureLabel*, *Input.FeatureType* and *Input.params*, as described in the article. For *Input.params*, except for three common parameters, *Input.params.NCluster*, *Input.params.maxiter*, *Input.params.tol*, there are several method-specific parameters. In the article, we have described SNMNMF-specific parameters. Here, we describe other methods specific parameters in detail.

For jNMF, there are two specific parameters:

- *Input.params.nloop*: The number of repeating times to run this algorithm. To obtain a robust and good solution, this algorithm is run for multiple times repeatedly, and the solution with the minimal objective function value is accepted. For example, we may set *Input.params.nloop* $= 50$.

- *Input.params.thrd_module*: A non-negative vector of size $1 \times (N + 1)$ to select features in md-modules. *Input.params.thrd_module*$(i + 1)$ is the threshold for selecting the i-th type of features in *Input.data* $(i = 1, ..., N)$. The first one is for selecting samples. The larger they are, the smaller number of features are selected. Users can set it based on the size of md-modules they prefer to identify. For example, we may set *Input.params.thrd_module = ones(1,N+1).*

For sMBPLS, there are:
- *Input.params.nfold*: A positive number used for n-fold cross-validation (CV) procedure. Generally, we set *Input.params.nfold* $= 5$ or 10. This method applies CV procedure to select a group of parameters from all the combinations of these parameter lists described below.
- *Input.params.thrXYr_list*: A column vector with positive integers. They are candidates for threshold to select samples in md-modules. For example, we may set *Input.params.thrXYr_list* = [20;30].
- *Input.params.thrXc_list, Input.params.thrYc_list*: Two row vectors of size $1 \times N, 1 \times M$ with positive integers to control the sparsity degree of weight variables for input data X, Y, respectively. For example, we may set *Input.params.thrXc_list* $=$ repmat$(\{[20; 30]\}, 1, N)$, *Input.params.thrYc_list* $=$ repmat$(\{[20; 30]\}, 1, M)$, where $N =$ *size(Input.XBlockInd,1)*, $M =$ *size(Input.YBlockInd,1).*

For SNPLS, there are:
- *Input.params.nfold*: It is the same as that in sMBPLS.
- *Input.params.thrXc_list, Input.params.thrYc_list*: They have the same meaning as those in sMBPLS for the situation of $N = 1$, $M = 1$. Thus, they are defined as column vectors. For example, we may set *Input.params.thrXc_list* $= [0.01; 0.03; 0.05]$, *Input.params.thrYc_list* $= [0.1; 0.3; 0.5]$.
- *Input.params.thrXNet_list, Input.params.thrYNet_list*: Two column vectors with non-negative values. They have the similar function with *Input.params.thrNet11* in SNMNMF. *Input.params.thrXNet_list, Input.params.thrYNet_list* are respectively for the networks within the features in input data X and response data Y. For example, we may set *Input.params.thrXNet_list* $= [1; 5]$; *Input.params.thrYNet_list* $= [1; 5]$.
- *Input.params.thrd_module*: It is a non-negative matrix of size 3×2. The first column provides thresholds for selecting samples, features in X and Y, respectively. The second column provides the minimal percentage of selected members when no features are selected under the given thresholds. The larger the thresholds are, the smaller number of features are selected. For example, we may set *Input.params.thrd_module* $= [1, 0.5; 1, 0.5; 1, 0.5]$.

With this data structure, MIA is able to partition *Input.data* into corresponding data matrices as input for each method automatically.

Here, we provide an example of input data for SNMNMF method (Figure 1).

4 Output results

Given input data and desired method, MIA automatically performs all computations and saves all the results in a specific folder named *'***_Results'*, where *** represents the pre-selected method name. Referring to the results, there are four parts, including a MATLAB data file named *'***_Results.mat'* (Figure 3c), some figures (Figure 2b, Figure 4, Figure 5), two text files (Figure 3d), and some folders named *'***Lists'* (Figure 3a).

Referring to output figures, there are some differences between NMF-class and PLS-class methods. For jNMF and SNMNMF, we provide the box-plots of sample-wise correlations

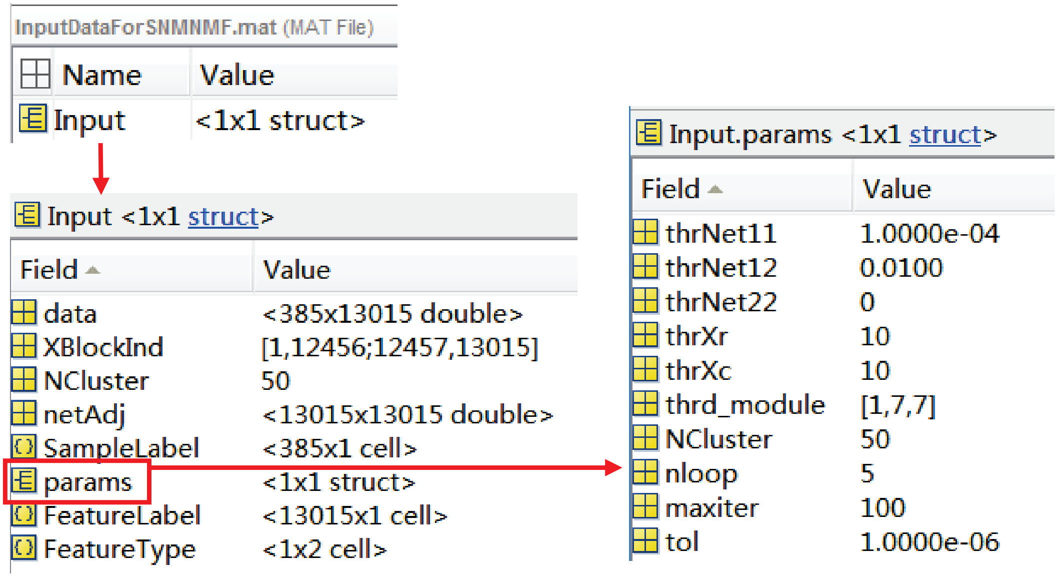

Figure 1: Illustration of an example of input data for *SNMNMF*.

of the original data and corresponding reconstructed data by factorized matrices, named as *'Correlations_between_original_space_and_reconstructed_space.fig'* (Figure 4). The large medians indicate that the dimension reduction technique captures the major information hidden in the original data. For sMBPLS and SNPLS, we also provide heatmaps of reordered input data, named *'Reordered_data_*.fig'*, where * denotes the index of identified md-module (Figure 5). The reordered data are obtained by reordering rows and columns of original input data matrices such that the embedded module could be observed in left-top, left-bottom, right-top and right-bottom corners in the heatmaps.

5 Demo

Here, we provide an example to introduce how to use MIA package. Use the data we demonstrate in section 3 (Figure 1), and select 'SNMNMF' method. Input the command in the window of MATLAB:

>> *MIA(Input, 'SNMNMF')*;

All the results are saved in the directory *'MIA/SNMNMF/SNMNMF_Results/'*. In this folder, we obtain these files as shown in Figure 2, Figure 3 and Figure 4.

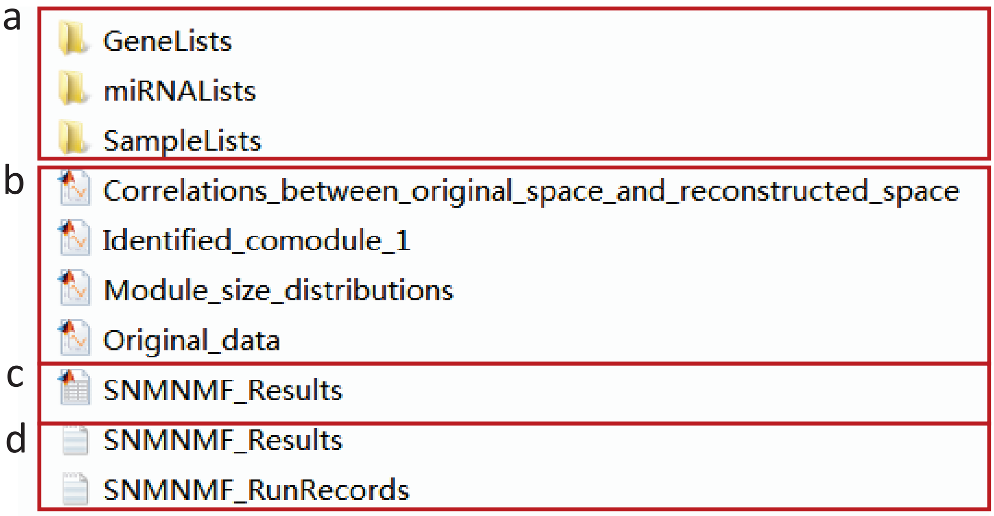

Figure 2: An overview for outputs of *SNMNMF*. The details about each part are shown in Figure 3 and Figure 4.

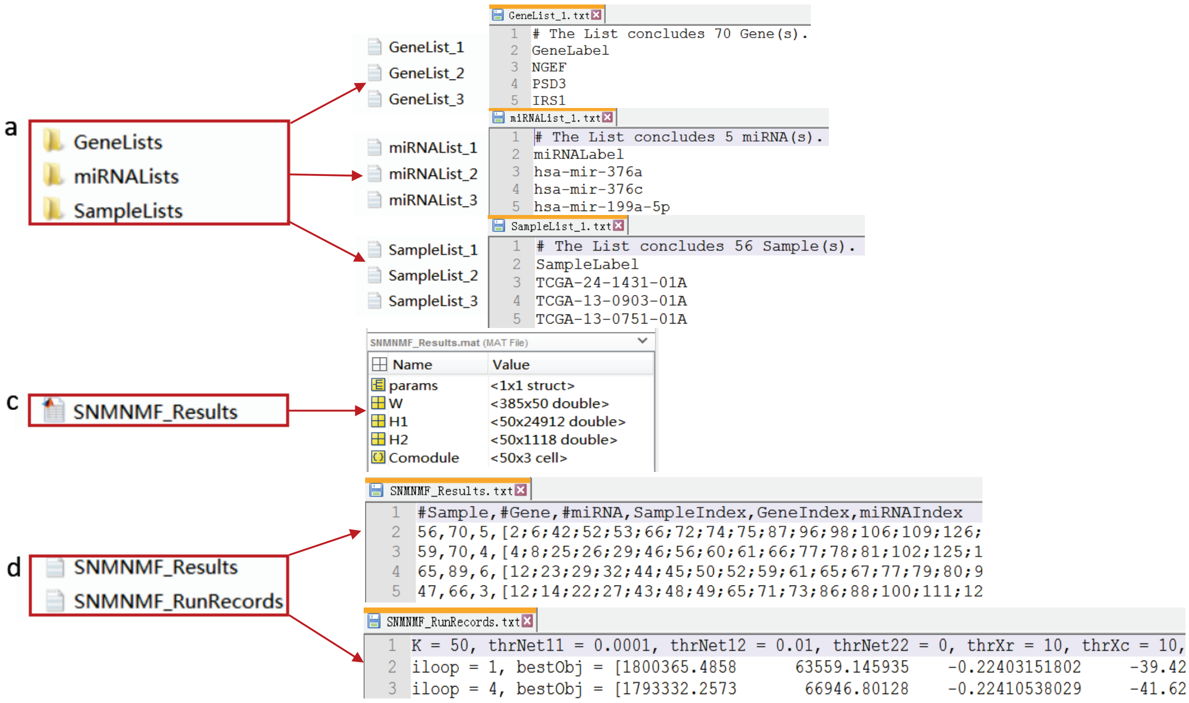

Figure 3: The details about output files shown in Figure 2. (a) In each folder, there are a number of text files, each of which records one type of components in one identified co-module. (c) A MATLAB data file storing the computation results, including factored matrices W, H_1, H_2, 50 identified co-modules and parameters used in this method. (d) The first text file records the feature indexes of all the identified md-modules, in which the first three columns are respectively the numbers of samples, genes, microRNAs in one identified co-module, and the next three columns show the indexes of selected samples, genes and microRNAs included in the square brackets. The second text file records the used parameters in the algorithm (the first row) and the objective function values during multiple-round running (the rest rows). It just keeps track of the results if the objective value is smaller than that of a previous round. *bestObj* stores the values of all the terms in the objective function in the '*iloop*'-th round, and *sum_Obj* equals the sum of these terms.

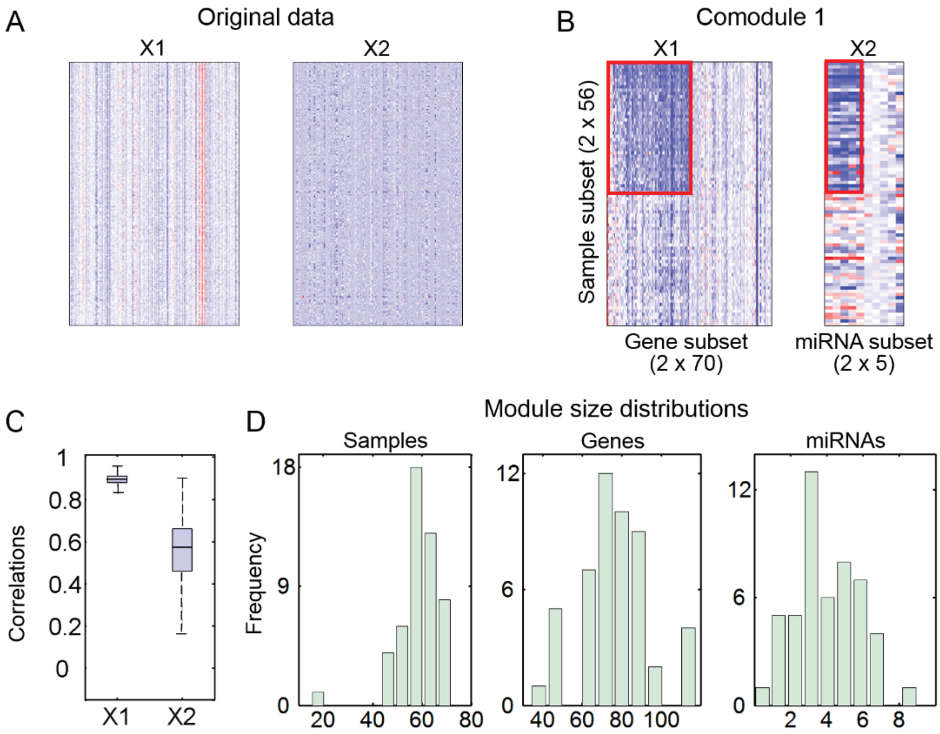

Figure 4: An example for output figures in Figure 2b. (A) Heatmaps of input data. (B) An example for heatmaps of identified md-modules (circled in red lines) and randomly selected features for comparison. (C) Sample-wise correlations between the original data and corresponding reconstructed data by factorized matrices. (D) Size distribution for each type of components in md-modules.

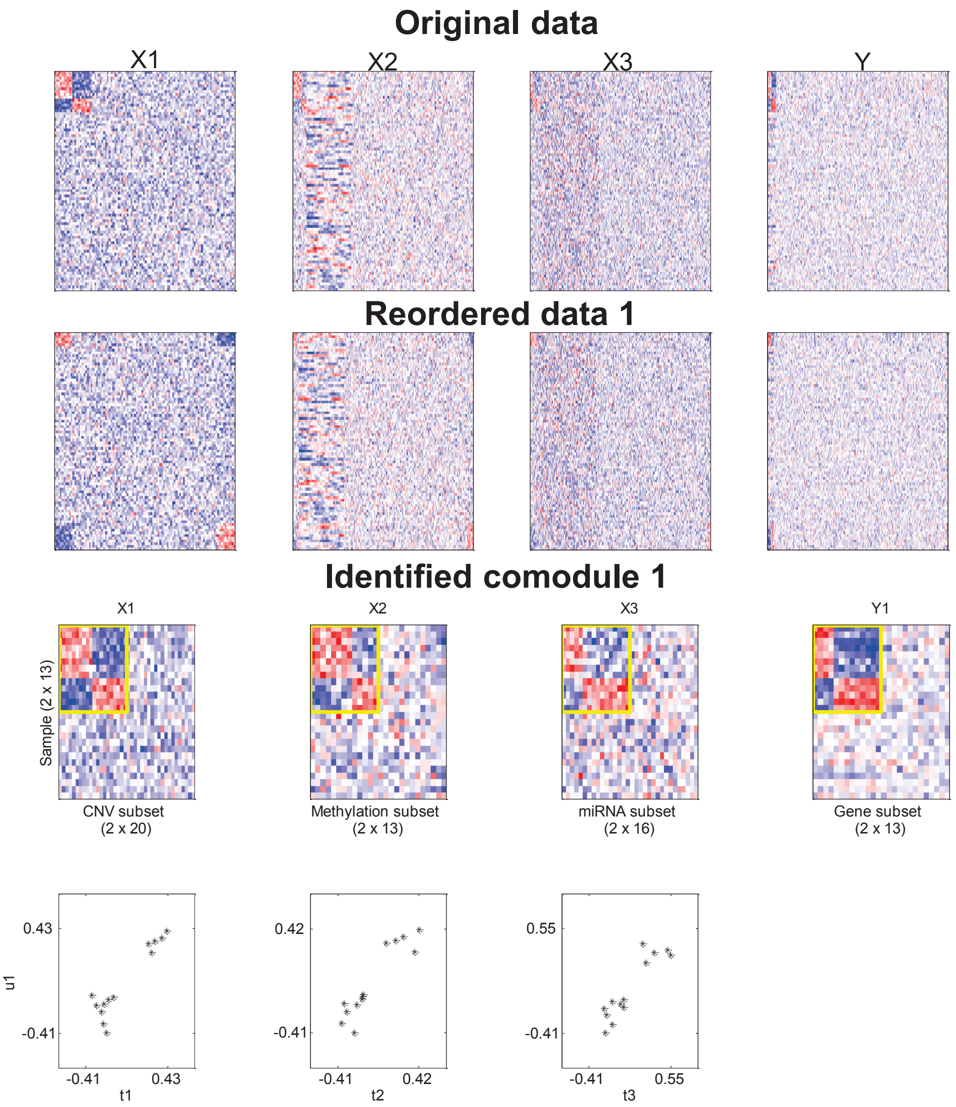

Figure 5: An example for output figures of sMBPLS. Heatmaps for the original input data and reordered input data based on the first identified co-module such that its members are located in the four corners in the heatmaps. In co-module 1, its members are in the left-top corner and the same number of features are randomly selected for comparison. Scatter plots show high correlations between latent vectors u_i for X_i and t for Y.

6 Simulation study

In order to clearly demonstrate the differences between NMF-based methods and PLS-based methods, we construct a set of simulated data and embed five co-modules in them with distinct characteristics. We then apply jNMF and sMBPLS as representatives of the two types of methods to simulated data, respectively. By comparing their performance, we could get direct understanding for what patterns the two types of methods tend to discover, respectively.

Without the loss of generality, we only simulate two data matrices X and Y which are adaptable for all the four methods in MIA. We set $p = 500$, $n = 1000$ for X and $m = 500$ for Y, that is, $X \in \mathbb{R}^{500\times1000}$ and $Y \in \mathbb{R}^{500\times500}$. We construct X as follows:

We firstly simulate the background signals of X. $X_j^{(0)}$ is the j-th column of matrix $X^{(0)}$ which is generated by several hidden components $U^{(k)}$ such that

$$X_j^{(0)} = \begin{cases} U^{(1)}, & \text{if } 1 \le j \le 200, \\ U^{(2)}, & \text{if } 201 \le j \le 400, \\ U^{(3)}, & \text{if } 401 \le j \le 600, \\ U^{(4)}, & \text{if } 601 \le j \le 810, \\ U^{(5)}, & \text{if } 811 \le j \le 1000, \end{cases}$$

where the i-th element $u_i^{(k)}$ of column vector $U^{(k)}$ is defined below:

$$U^{(1)} = (u_i^{(1)}) = 1.3 + 0.2I(u \le 0.5), 1 \le i \le 500,$$

$$U^{(2)} = (u_i^{(2)}) = 1.2 + 0.2I(u \le 0.5), 1 \le i \le 500,$$

$$U^{(3)} = (u_i^{(3)}) = 1 + 0.5I(u \le 0.5), 1 \le i \le 500,$$

$$U^{(4)} = (u_i^{(4)}) = 1.3 + 0.5I(u \le 0.1), 1 \le i \le 500,$$

$$U^{(5)} = (u_i^{(5)}) = 1 + I(u \le 0.2), 1 \le i \le 500,$$

where $u \sim \text{Unif}(0, 1)$.

Then we set $X = X^{(0)}$ and construct five modules sequentially:

$$X_{ij} = \begin{cases} 2.8, & \text{if } 1 \le i \le 50, 1 \le j \le 100, \\ 4, & \text{if } 51 \le i \le 100, 1 \le j \le 100, \\ 2.8, & \text{if } 1 \le i \le 25 \text{ and } 51 \le i \le 75, 101 \le j \le 200, \\ 4, & \text{if } 26 \le i \le 50 \text{ and } 76 \le i \le 100, 101 \le j \le 200. \end{cases}$$

$$X_{ij} = \begin{cases} -0.4 + 0.2 \times \lfloor \frac{i-111}{10} \rfloor, & \text{if } 111 \le i \le 150, 201 \le j \le 400, \\ 3 + 0.2 \times \lfloor \frac{i-151}{10} \rfloor, & \text{if } 151 \le i \le 200, 201 \le j \le 400. \end{cases}$$

where $\lfloor x \rfloor$ denotes rounding x to the nearest integer less than or equal to x.

$$X_{ij} = \begin{cases} -1.5 + 0.5I(u \le 0.5), & \text{if } 191 \le i \le 300, 401 \le j \le 450, \\ 3.6 + 0.4I(u \le 0.5), & \text{if } 191 \le i \le 300, 451 \le j \le 600. \end{cases}$$

$$X_{ij} = 3 + I(u \le 0.3), \text{if } 281 \le i \le 350, 581 \le j \le 770.$$

$$X_{ij} = 2.8 + u, \text{if } 416 \le i \le 490, 811 \le j \le 1000.$$

Here $u \sim \text{Unif}(0, 1)$.

Next, we simulate background signals of $Y^{(0)}$ by $Y^{(0)} = XB$ and

$$B_{ij} = \begin{cases} 0.005, & \text{if } 1 \leq i \leq 200, 1 \leq j \leq 100, \\ 0.004, & \text{if } 201 \leq i \leq 400, 101 \leq j \leq 200, \\ \frac{N(1,1)}{200}, & \text{if } 401 \leq i \leq 810, 201 \leq j \leq 400, \\ 0.004, & \text{if } 811 \leq i \leq 1000, 401 \leq j \leq 500, \\ 0, & \text{otherwise,} \end{cases}$$

where $N(1, 1)$ represents a random value from a normal distribution with mean 1 and standard deviation 1.

Similarly, based on $Y^{(0)}$, we set $Y = Y^{(0)}$ and add five module signals sequentially:

Y(1:100, 1:50) = X(1:100, 1:200)$B^{(1)}$, where $B^{(1)}_{ij} = 0.007, 1 \leq i \leq 200, 1 \leq j \leq 50;$ Y(1:100, 51:100) = X(1:100, 1:200)$B^{(2)}$, where $B^{(2)}_{ij} = -0.0035, 1 \leq i \leq 200, 1 \leq j \leq 50;$ $Y_{ij} = 3.5 + u, 1 \leq i \leq 100, 201 \leq j \leq 230$ and $u \sim$ Unif$(0, 1)$;

Y(111:200, 101:200) = X(111:200, 201:400)$B^{(3)}$, where $B^{(3)}_{ij} = 0.007, 1 \leq i \leq 200, 1 \leq j \leq 100$;

Y(201:300, 201:300) = X(201:300, 401:600)$B^{(4)}$, where $B^{(4)} = (\beta, ..., \beta) \in \mathbb{R}^{200\times100}$, and β is the random permutation of vector
$10^{(-3)} \times (\underbrace{8, 9, 10, 11, 12}, \underbrace{8, 9, 10, 11, 12}, \cdots, \underbrace{8, 9, 10, 11, 12})^T \in \mathbb{R}^{200\times1}$;

Y(281:350, 291:360) = X(281:350, 581:770)$B^{(5)}$, where $B^{(5)}_{ij} = 0.008, 1 \leq i \leq 190, 1 \leq j \leq 70;$ Y(281:350, 361:400) = X(281:350, 581:770)$B^{(6)}$, where $B^{(6)}_{ij} = -0.004, 1 \leq i \leq 190, 1 \leq j \leq 40$;

Y(416:490, 401:490) = X(416:490, 811:1000)$B^{(7)}$, where $B^{(7)}_{ij} = 0.008, 1 \leq i \leq 190, 1 \leq j \leq 90$.

Finally, we set $X^* = X + \Xi_x$, where $\Xi_x \sim N(0, \sigma_x^2 \mathbf{I}_{1000})$ (σ_x=3:0.5:6) and $Y^* = Y + \Xi_y$, where $\Xi_y \sim N(0, \sigma_y^2 \mathbf{I}_{500})$ (σ_y=3:0.5:6).

Besides, since the signals for parts X(191:300, 401:450) and Y(1:100, 51:100), Y(281:350, 361:400) are rather lower than other parts, we reduce the added noise to one quarter for the three parts in case of covering the embedded module signals. We show an example for simulated data in Figure 6.

Besides, since the signals for parts X(191:300, 401:450) and Y(1:100, 51:100), Y(281:350, 361:400) are rather lower than other parts, we reduce the added noise to one quarter for the three parts in case of covering the embedded module signals. We show an example for simulated data in Figure 6.

We expect to use the simulated data to demonstrate the differences between NMF-based (jNMF and SNMNMF) and PLS-based (sMBPLS and SNPLS) methods when identifying the embedded patterns in data matrices. Generally, NMF prefers to discover local patterns with much high absolute signals across the same set of samples whereas PLS emphasizes regression analysis to find out the features with relatively high positive or negative correlations. Based on the simulated data structure described above and the different preferences of these two types of methods, we respectively provide the module members that jNMF and sMBPLS should identify, denoted as $G^{(1)}$ and $G^{(2)}$ (Table 1 and 2). We name $G^{(1)}$ as NMF-based standard and $G^{(2)}$ as PLS-based standard. If the co-modules identified by jNMF approximate to $G^{(1)}$ more closely than those by sMBPLS and the co-modules by sMBPLS are more close to $G^{(2)}$ than jNMF, it will verify the differences between these two types of methods. Here we use the relevance score to measure the degree of similarity between the k-th real co-module $G^{(i)}_k$ and the identified one

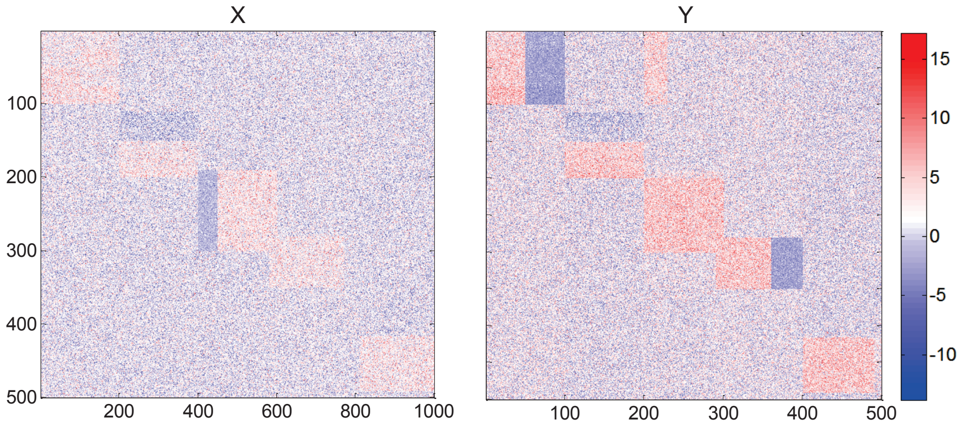

Figure 6: An example for simulated data matrices X and Y.

$M_k (k = 1, 2, 3, 4, 5)$ by jNMF and sMBPLS:

$$\text{relevance}(M_j, G^{(i)}) = \max_k s(M_j, G_k^{(i)}), s(M_j, G_k^{(i)}) = \frac{|G_k^{(i)} \cap M_j|}{|G_k^{(i)} \cup M_j|}$$

where $|A \cap B|$ is the number of members of their intersection and $|A \cup B|$ is the number of members of their union. For each level of data noise, we repeat the simulation procedure for 50 times and compute the relevance scores for five co-modules respectively under two golden standard sets $G^{(1)}$ and $G^{(2)}$ (Figure 7–11).

Table 1: The golden standard for co-module components that NMF-based methods should identify.

Co-module ID	Sample	X	Y
1	1:100	1:200	1:50, 201:230
2	151:200	201:400	101:200
3	201:300	451:600	201:300
4	281:350	581:770	291:360
5	416:490	811:1000	401:490

Sample: row indexes in simulated data. X, Y: column indexes in simulated matrices X and Y. 1:100 means all the numbers from 1 to 100.

Table 2: The golden standard for co-module components that PLS-based methods should identify.

Co-module ID	Sample	X	Y
1	1:100	1:200	1:100
2	111:200	201:400	101:200
3	201:300	401:600	201:300
4	281:350	581:770	291:400
5	416:490	811:1000	401:490

These figures show that co-modules identified by jNMF are more similar to $G^{(1)}$ than those by sMBPLS. It verifies that jNMF indeed tend to discover local patterns with high signals. For example, for the first embedded module in the simulated data Y (i.e., features from the first to 100th column and from 201st to 230th column), the first 100 components are highly positively or negatively correlated with each other. In the first 100 components, the last 50 ones have rather low signals. The other 30 components (from the 201st to 230th column) have high signals

with similar magnitude to the first 50 components, but they have weak correlations with the first 100 components. It shows that jNMF identifies features from the first to 50th column and from 201st to 230th column, whereas sMBPLS discovers those from the first to 100th column. Meanwhile, co-modules by sMBPLS are more similar to $G^{(2)}$ than those by jNMF, which suggests sMBPLS could identify the highly correlated components, not only for samples (e.g., the second co-module) but also for features in X (e.g., the third co-module) and Y (e.g., the fourth co-module) even with slightly low signals. In such situations, jNMF often disregards them.

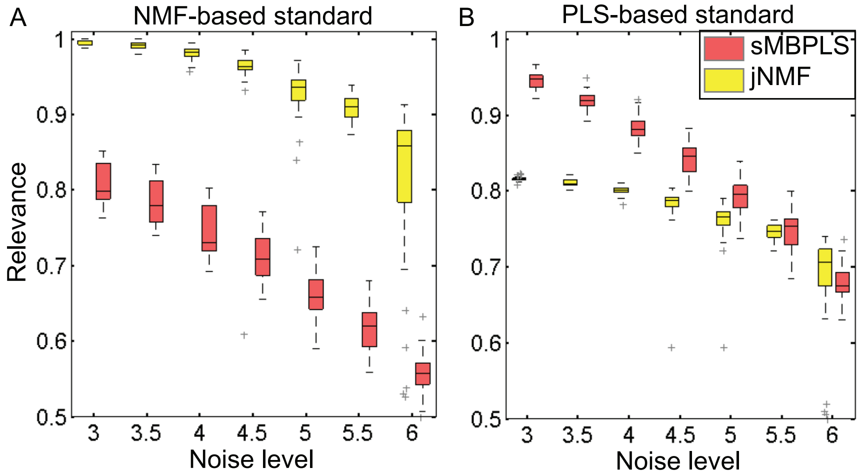

Figure 7: Performance comparison of jNMF and sMBPLS in terms of relevance scores for the first identified co-module under two golden standards. Here, we apply jNMF and sMBPLS to 50 sets of simulated data matrices for each level of data noise, respectively.

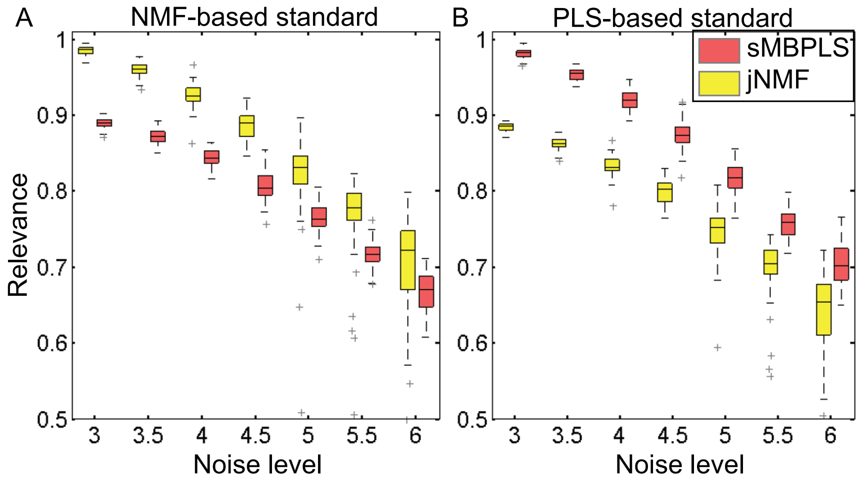

Figure 8: It is for the second co-module with similar setting in Figure 7.

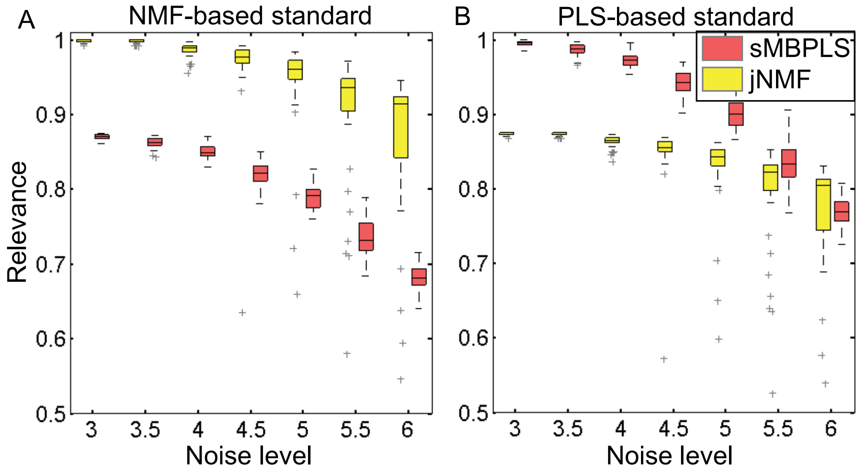

Figure 9: It is for the third co-module with similar setting in Figure 7.

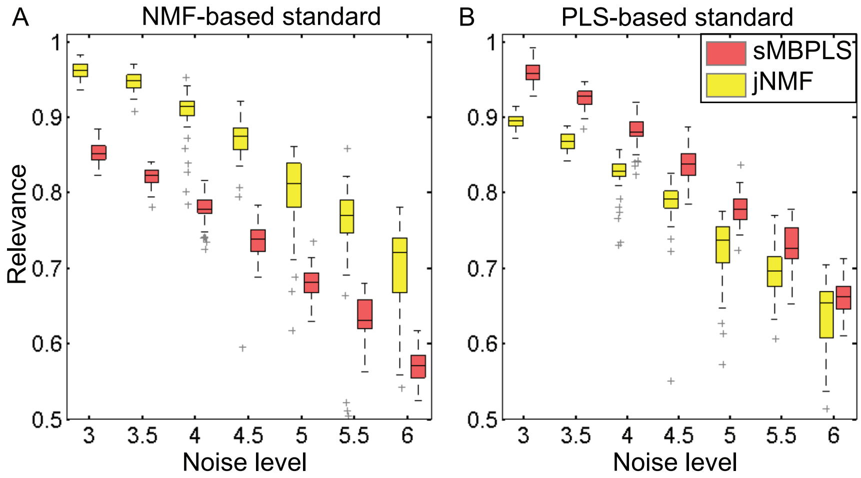

Figure 10: It is for the fourth co-module with similar setting in Figure 7.

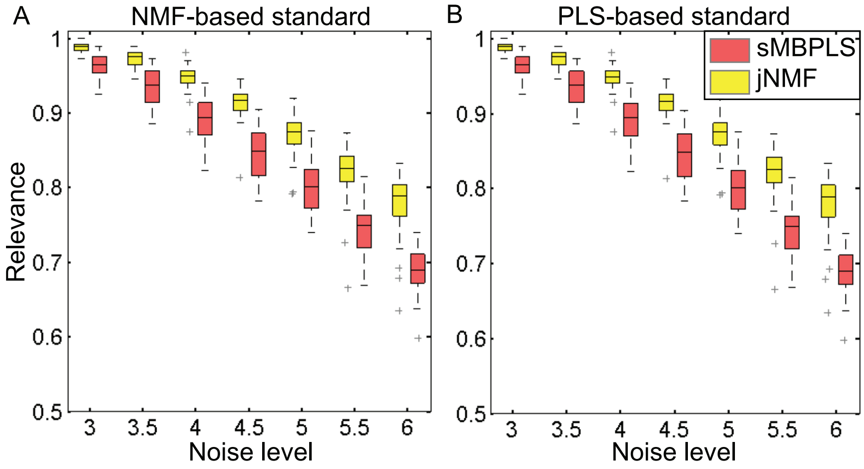

Figure 11: It is for the fifth co-module with similar setting in Figure 7.

References

Chen, J. and Zhang, S. (2016) Integrative analysis for identifying joint modular patterns of gene-expression and drug-response data. *Bioinformatics*, **32**, 1724-1732.

Li, W. *et al.* (2012) Identifying multi-layer gene regulatory modules from multi-dimensional genomic data. *Bioinformatics*, **28**, 2458-2466.

Zhang, S. *et al.* (2011) A novel computational framework for simultaneous integration of multiple types of genomic data to identify microRNA-gene regulatory modules. *Bioinformatics*, **27**, i401-i409.

Zhang, S. *et al.* (2012) Discovery of multi-dimensional modules by integrative analysis of cancer genomic data. *Nucleic Acids Res*, **40**, 9379-9391.
