# Supplementary material for: Matrix Integrative Analysis (MIA) of Multiple Genomic Data for Modular Patterns
Source: Front Genet. 2018 May 29;9:194. doi: 10.3389/fgene.2018.00194 (PMC5992392; doi:10.3389/fgene.2018.00194)
Supplement: Supplementary file 2 [file Presentation_2.PDF]

Package ‘MIA’

Version 1.0
Date 2016-06-12
Title Matrix Integration Analysis
Author Jinyu Chen <chjy@amss.ac.cn>, Shihua Zhang <zsh@amss.ac.cn>
Depends MATLAB ($\geq$ R2013a)
Description Detecting multi-dimensitional modules (md-modules) in diverse genomics data as well as molecular network data using the methods in MIA package.
URL http://page.amss.ac.cn/shihua.zhang/software.html

Index

Index2LabelForModuleContent, 7, 11, 18, 23

jNMF_algorithm, 4
jNMF_comodule, 4
jNMF_module, 5
jNMF_plot_correlation, 6
jNMF_plot_distribution, 7
jNMF_plot_X, 5
jNMF_PrepData, 3

meanc, 12

newMatrix, 16

OutputModule2TXT, 7, 11, 18, 23

PLS, 22

Run_jNMF, 3
Run_sMBPLS, 12
Run_SNMNMF, 8
Run_SNPLS, 19

sMBPLS_algorithm, 14
sMBPLS_comodule, 12
sMBPLS_getCVscore, 16
sMBPLS_params, 15
sMBPLS_plot_distribution, 18
sMBPLS_plot_results, 17
sMBPLS_plot_XY, 17
sMBPLS_select_param, 15
SNMNMF_algorithm, 10
SNMNMF_comodule, 9
SNMNMF_module, 10
SNMNMF_plot_correlation, 11
SNMNMF_plot_distribution, 11
SNMNMF_plot_results, 11
SNMNMF_plot_X, 10
SNMNMF_PrepData, 9
SNPLS_algorithm, 20
SNPLS_comodule, 19
SNPLS_getCVscore, 21
SNPLS_params, 20
SNPLS_plot_distribution, 23
SNPLS_plot_results, 23
SNPLS_plot_XY, 22
SNPLS_select_param, 21

thresholding, 22

variable_sparse, 16

Here, we provide a guide for MIA package. It describes all the MATLAB functions in MIA in detail. For each method, these MATLAB functions mainly perform the tasks including implementing a specific algorithm, drawing figures and outputting text files about the identified md-modules.

1 Implementation

1.1 Users with a MATLAB license

Requirements: It works in MATLAB R2013a or later in Windows (64-bit).

1) Download MIA pacakge in the website: http://page.amss.ac.cn/shihua.zhang/software.html.

2) Unzip the package into a specific directory (e.g., *'D:/'*), and set the work path of MATLAB (e.g., *'D:/MIA/'*).

3) Load the data. In the MIA package, there is a folder named *'InputData'* storing the demo input data for these four methods. For example, we load the data as the input for SNMNMF.

   >> *load('InputData/InputDataForSNMNMF.mat','Input')*;

4) Run the main function *MIA.m* with a desired method. For example, we select SNMNMF for analyzing the loaded data.

   >> *MIA(Input, 'SNMNMF')*;

Then, MIA automatically performs all computations and saves all the results into the path *'MIA/SNMNMF/SNMNMF_Results/'*.

1.2 Users without a MATLAB license

Requirements: It works in Windows (64-bit) operating system. And the Windows 64-bit version of the MATLAB Runtime for R2015b is required to install which is available from the MathWorks Web site by navigating to http://www.mathworks.com/products/compiler/mcr/index.html.

1) Prepare the input data and store them in the path *'D:/MIA/InputData/'*. In this folder, we provide the input data for each method as examples. For each method, there are two Excel files (one is for input data matrices and another one is for input parameters), each of which includes several sheets. Users need to arrange their data in the same way as those example Excel files in this folder we provided. Note that, each sheet is renamed as the corresponding variable name as described in our manuscript.

2) Open the Command Prompt (*cmd.exe*). Set the current path as where the MIA package is located, e.g., *'D:/MIA'*.

3) Produce MATLAB data files (**.mat*) for selected method by running *PreInputData.exe*. Taking SNMNMF for example, type the command as below:

   PreInputData.exe *./InputData/DataForSNMNMF.xlsx* *./InputData/ParametersForSNMNMF.xlsx* *./InputData/InputDataForSNMNMF.mat* *SNMNMF*

   Note that, the first argument of *PreInputData.exe* is the name of Excel file storing input data matrices; the second one is the name of Excel file recording all the input parameters; the third one is the output file name, given by users. Its output is a MATLAB-data file, saving all the input data for some method; the last one is the name of what the produced

input data is used for. The produced new data files (e.g., *'InputDataForSNMNMF.mat'*) are saved in the directory *'D:/MIA/InputData/'*.

4) Run *MIA.exe*. Type the command as below:

   MIA.exe *./InputData/InputDataForSNMNMF.mat* *SNMNMF*

   The first argument of *MIA.exe* is the input data file name and the second one is the selected method name. For each method, the results are saved their own directory. For example, the results of running SNMNMF are saved in *'./MIA/SNMNMF/SNMNMF_Results/'*.

2 jNMF

jNMF (**j**oint **N**on-negative **M**atrix **F**actorization) enables users to simultaneously factor two or more types of genomic data sharing the same set of samples. Here, we adopt multiplicative update algorithm to solve the following problem:

$$\min_{W,H_i} \sum_{i=1}^{N} \|X_i - WH_i\|_F^2, \quad \text{s.t. } W \geq 0, H_i \geq 0, i = 1, ..., N.$$

2.1 Algorithm

Run_jNMF	*The main function for jNMF.*

Description

This is the main function for jNMF, which integrates all the related functions to achieve it.

Usage

Run_jNMF(Input);

Arguments

Input	A structure variable. The details about its construction can be found in Section 6.

Output

It saves all the results in the directory *'./MIA/jNMF/jNMF_Results/'*.

jNMF_Results.mat	The essential computed variables.
jNMF_RunRecords.txt	The updated values of the objective function in each round of running.
jNMF_Results.txt	The numbers and indexes of features separated in comma. Each row records the information for each identified md-module.
Several folders	Each folder contains the lists of all the identified md-modules for one type of features as shown in Figure 2a and Figure 3a.
Several figures	As shown in Figure 2b and Figure 4.

jNMF_PrepData	*Preprocess the input data.*

Description

This function is used to preprocess the input data to ensure its non-negativity.

Usage

[X, isdouble] = jNMF_PrepData(OX);

Arguments

OX	A matrix.

Output

X	A non-negative matrix, the transformation of data *OX*.
isdouble	A binary variable (1 indicates changes have been made; 0 is for no change, that is, $X = OX$).

---

jNMF_comodule *Obtain the md-modules.*

Description

This function outputs the optimal factorization results through running jNMF for multiple times, then identify md-modules based on the factorized matrices W, H_i ($i = 1, 2, ...$).

Usage

[W, H, Comodule, params] = jNMF_comodule(Input, params);

Arguments

Input	A structure variable including two components:
Input.data	A non-negative matrix combing N data blocks sequentially to be factorized, such as *Input.data* $= [X_1, X_2, ..., X_N]$.
Input.XBlockInd	A matrix of size $N \times 2$. The two elements of the ith row give the start and end column indexes in *Input.data* for data matrix X_i $(i = 1, ..., N)$.
params	A structure variable including six components:
params.isdouble	A vector of size $N \times 1$. The ith element indicates whether the ith data matrix is transformed to ensure its non-negativity (0 for no change, and 1 for change).
params.K	The number of md-modules users prefer to identify.
params.nloop	The repeating times of jNMF. To ensure the robust of this method, this function repeats the algorithm for 'params.nloop' times.
params.maxiter	The maximal number of iterations for this algorithm.
params.tol	The precision for convergence of algorithm.
params.thrd_module	A positive vector of size $1\times(N+1)$. Thresholds for selecting features in N data blocks. The first one is to select samples.

Output

W, H	Factorization results such that *Input.data* $\approx$ *WH*. *W* is the basis matrix of size $m \times K$ and *H* is the weight matrix of size $K \times n$, where $K =$ *params.K*.
Comodule	Identified md-modules recorded in a $(K\times(N+1))$ cell array. *Comodule*$\{$*i,j*$\}$ records selected feature indexes of the jth type of variables in the ith identified md-module. The first column is for selected samples.
params	Compared to *'params'* as input, there are something new added in it, including
params.records	A (*nloop*$\times$1) cell array. *params.records*$\{i\}$ is a (*iter*$\times$ $(N + 1)$) vector, where each row records the values of all the terms in the objective function and the sum of them in each iteration.
params.iterNumList	A (*nloop*$\times$1) vector, where each element is the number of iterations for each round of running.

---

jNMF_algorithm *jNMF algorithm.*

Description

This is jNMF algorithm.

Usage

[W, H, TerminalObj, iter] = jNMF_algorithm(X, XInd, params);

Arguments

X	A non-negative input matrix of size $m \times n$ combing N data matrices sequentially to be factorized (e.g., $X = [X_1, X_2, ..., X_N]$).
XInd	A matrix of size $N \times 2$. The two elements of the ith row give the start and end column indexes in *X* for data matrix X_i $(i = 1, ..., N)$.
params	A structure variable including *params.K, params.maxiter, params.tol* defined the same as that in the function '*jNMF_comodule*'.

Output

W, H	Factorization results like those in function '*jNMF_comodule*'.
iter	The number of iterations when the algorithm stops.
TerminalObj	A (*iter* $\times (N + 1)$) matrix in which each row records the values of all the terms in the objective function and the sum of them in each iteration.

---

jNMF_module *Identify md-modules from factorized matrices.*

Description

Based on the factorized matrix W or H_i, identify module members for each type of features.

Usage

module = jNMF_module(H, t, isdouble);

Arguments

H	A $(K \times m)$ non-negative matrix used for module identification.
t	A threshold value for selecting features.
isdouble	A binary variable (1 is for the number of features in matrix *H* is double than that of the original ones, and 0 is for no change).

Output

module	A $(K \times 1)$ cell array. modulei,1 contains the feature indexes of the ith module.

2.2 Output figures

---

jNMF_plot_X *Provide the heatmaps of the original input matrices.*

Description

Draw the heatmaps of the original input matrices $(X_1, X_2, ..., X_N)$.

Usage

jNMF_plot_X(X, XInd, fig, figure_title, colormap_type);

Arguments

X	The input matrix combing N data matrices to be factorized (e.g., $X = [X_1, X_2, ..., X_N]$).

XInd	A matrix of size $N \times 2$. The two elements of the ith row give the start and end column indexes in *X* for data matrix X_i $(i = 1, ..., N)$.
fig	A positive integer for figure index.
figure_title	A string for the title of figure.
colormap_type	A string for the colormap of heatmaps. Options includes 'blue-yellow', 'green-red', 'yellow', 'blue-white-red', 'default'.

Output

The heatmaps for all the input data matrices as shown in Figure 4.

---

jNMF_plot_results — *Show the heatmaps of certain identified md-modules.*

Description

Show the heatmaps of a selected identified md-module (circled in yellow lines).

Usage

jNMF_plot_results(X, XInd, FeatureType, fig, figure_title, colormap_type, vectorForRank);

Arguments

X, XInd, fig, figure_title, colormap_type

They have the same definitions with those in function *jNMF_plot_X*.

FeatureType	A $(1 \times N)$ cell array. *FeatureType*$\{i\}$ records the name of ith type of features (e.g., *FeatureType* = $\{$*'Gene expression','microRNA expression','CNV'*$\}$).
vectorForRank	A structure variable containing four components:

vectorForRank.w, vectorForRank.h

Two vectors for the selected md-module. For example, if one wants to demonstrate the ith md-module, *vectorForRank.w* is the ith column of basis matrix W and *vectorForRank.h* is the ith row of weight matrix H.

vectorForRank.comodule

A $(1\times(N + 1))$ cell array. *vectorForRank.comodule*$\{i\}$ records the ith feature indexes of the selected md-module.

vectorForRank.hInd	Similar with the input variable *'XInd'* to *'X'*, it records the indexes for *'vectorForRank.h'*.

Output

The heatmaps for a selected md-module to demonstrate its patterns as shown in Figure 4.

---

jNMF_plot_correlation — *Demonstrate the correlations between the original data and reconstructed data.*

Description

Demonstrate the correlations between the original data X_i and reconstructed data *newX*$_i$ = WH_i using boxplots.

Usage

corrMat = jNMF_plot_correlation(X, newX, XInd, newXInd, fig, figure_title);

Arguments

X, XInd, fig, figure_title

They have the same definitions with those in function '*jNMF_plot_X*'.

newX	The reconstructed matrix combing N data matrices sequentially, that is, *newX* $= [WH_1, WH_2, ..., WH_N]$.

newXInd — Similar with the input variable *'XInd'* to *'X'*, it records the indexes for matrix *newX*.

Output

corrMat — A matrix of size $m \times N$, where m is the number of samples and N is the input data matrices, respectively. *corrMat(i,j)* records the correlation between the ith rows of the original data (X_j) and reconstructed data (WH_j).

Boxplots for input data matrices $X_1, X_2, ..., X_N$ as shown in Figure 4.

---

jNMF_plot_distribution — *Demonstrate the module size distributions.*

Description

This function provides the histograms for the size distributions of $(N + 1)$ types of features in the identified md-modules.

Usage

jNMF_plot_distribution(nSample, XInd, Comodule, FeatureType, fig, figure_title);

Arguments

XInd, FeatureType, fig, figure_title
They are the same as those in function *'jNMF_plot_results'*.

nSample — The number of samples.

Comodule — It is the same as that in function *'jNMF_comodule'*.

Output

Histograms for the size distributions of $(N+1)$ types of features in the identified md-modules as shown in Figure 4.

2.3 Output into text files

Index2LabelForModuleContent — *Output the identified md-modules into text files.*

Description

Output a number of text files, each of which records the selected feature names.

Usage

Index2LabelForModuleContent(ModuleIndex, FeatureLabel, TypeName, ResultsFile);

Arguments

ModuleIndex — A $(K \times 1)$ cell array. *ModuleIndex*$\{i\}$ records the indexes of the ith identified module for one type of features.

FeatureLabel — A $(n \times 1)$ cell array recording all the names of one type of features.

TypeName — A string for the feature type (e.g., *TypeName = 'Gene expression'*).

ResultsFile — A string for the folder name to save these lists (e.g., *TypeName = 'Gene expression', ResultsFile = 'jNMF_results'*, all the identified gene lists are saved in the directory: *'./jNMF_results/GeneLists/'*.

Output

A number of text files (e.g., *GeneList_1.txt, GeneList_2.txt*, ...)

OutputModule2TXT — *Output the feature indexes of the identified md-modules.*

Description

Output the feature indexes of the identified md-modules into a text file.

Usage

OutputModule2TXT(Comodule, FeatureType, ResultsFile);

Arguments

Comodule	It is the same as that in function *'jNMF_comodule'*.
ResultsFile	A string for the name of this text file.

Output

A text file named as *jNMF_Results.txt* if define *ResultsFile = 'jNMF_Results'*, recording the number and indexes of selected features separated by comma.

3 SNMNMF

SNMNMF (**S**parse **N**etwork-regularized **M**ultiple **NMF**) can incorporate the interactions within ($A_{11} \in \mathbb{R}^{n_1 \times n_1}, A_{22} \in \mathbb{R}^{n_2 \times n_2}$) and between the two genomics variables ($A_{12} \in \mathbb{R}^{n_1 \times n_2}$) into the *jNMF* framework for pairwise case ($X_1 \in \mathbb{R}^{p \times n_1}, X_2 \in \mathbb{R}^{p \times n_2}$). Users can choose which networks they prefer to incorporate in the framework by setting the corresponding parameters. Besides, it also adds the sparsity constraints for basis matrix W and weight matrices H_1 and H_2. With the network-regularized constraints, *SNMNMF* makes the variables linked in these two networks more likely to be placed into the same module. With the sparsity constraints, it helps us choose key variables. These constraints both lead to the identified md-modules more biologically interpretable. This model is defined as follows:

$$\begin{aligned} \min_{W,H_i} \sum_{i=1}^{2} \|X_i - WH_i\|_F^2 - \sum_{1 \le i \le j \le 2} \lambda_{ij} Tr(H_i A_{ij} H_j^T) \\ +\gamma_1 \|W\|_F^2 + \gamma_2 (\sum_i \|h_i^{(1)}\|_1^2 + \sum_j \|h_j^{(2)}\|_1^2) \\ \text{s.t.} \quad W \ge 0, H_1 \ge 0, H_2 \ge 0. \end{aligned}$$

where $h_i^{(1)}$ is the ith column of H_1, and $h_j^{(2)}$ is the jth column of H_2.

3.1 Algorithm

Run_SNMNMF	*The main function for SNMNMF.*

Description

This is the main function for SNMNMF which integrates all the related functions to achieve it.

Usage

Run_SNMNMF(Input);

Arguments

Input	A structure variable (Section 6).

Output

It saves all the results in the directory *'./MIA/SNMNMF/SNMNMF_Results/'*, including *'SNMNMF_Results.mat'*, *'SNMNMF_RunRecords.txt'*, *'SNMNMF_Results.txt'*, several folders and figures, which record the similar contents with those in function *'Run_jNMF'* of jNMF as shown in Figure 2, Figure 3 and Figure 4.

SNMNMF_PrepData	*Preprocess the input data.*

Description

This function is used to preprocess the input data to ensure the non-negativity.

Usage

[newInput, isdouble] = SNMNMF_PrepData(Input);

Arguments

Input	A structure variable including three components:
Input.data	A matrix combing two data blocks sequentially to be factorized (e.g., *Input.data* $= [X_1, X_2]$).
Input.XBlockInd	A matrix of size 2×2. The two elements of the ith row give the start and end column indexes in *Input.data* for data matrix X_i $(i = 1, 2)$.
Input.netAdj	An adjacency matrix for the relationships between the features in *Input.data*, that is, Input.netAdj $= [A_{11}, A_{12}; A_{12}^T, A_{22}]$.

Output

newInput	A structure variable including
newInput.data	A non-negative matrix which is transformed from *Input.data*.
newInput.XBlockInd	A matrix of size 2×2 which is similar with *Input.XBlockInd*.
newInput.netAdj	An adjacency matrix transformed from *Input.netAdj*.
isdouble	A binary variable (1 indicates changes have been made, and 0 is for no change, that is, *Input* = *newInput*).

SNMNMF_comodule	*Obtain the md-modules.*

Description

This function computes the optimal factorization results through running SNMNMF for multiple times, then identify the md-modules based on the factorized matrices W, H_i.

Usage

[W, H1, H2, Comodule, params] = SNMNMF_comodule(Input, params);

Arguments

Input	A structure variable defined as that in function *'SNMNMF_PrepData'*, but the *Input.data* is a non-negative matrix.
params	A structure variable, except for *params.isdouble, params.K, params.nloop, params.maxiter, params.thrd_module,* defined similar as those in the function *'jNMF_comodule'* described above. There are five specific components, including
params.thrXr, params.thrXc	parameters referring to the basis matrix *W*-related term, the weight matrices H_i-related terms in the objective function to limit the growth of W, which make W, H_i sparse, respectively.
params.thrNet11, params.thrNet12, params.thrNet22	parameters referring to the network A_{11}, A_{12}, A_{22} related constraints in the objective function.

Output

W, H1, H2	Factorization results such that *Input.data* $\approx [WH_1, WH_2]$. W is

	the basis matrix of size $m \times K$, H_1 is the weight matrix of size $K \times n_1$ and H_2 is the weight matrix of size $K \times n_2$, where $K =$ *params.K*.
Comodule	Identified md-modules recorded in a $(K \times 3)$ cell array, which has the same definition as that in function *'jNMF_comodule'*.
params	Compared to 'params' as input, there are something new added in it, including *params.records* and *params.iterNumList* which are similar with those in the function *'jNMF_comodule'*, where $N = 2$.

SNMNMF_algorithm *SNMNMF algorithm.*

Description

This implements the SNMNMF algorithm.

Usage

[W, H1, H2, TerminalObj, iter] = SNMNMF_algorithm(X1, X2, A11, A12, A22, params);

Arguments

X1, X2	The non-negative input matrices.
A11, A12, A22	Adjacency matrices for the relationships within and between the features in X_1 and X_2.
params	A structure variable including *params.isdouble*, *params.K*, *params.thrd_module*, *params.thrXr*, *params.thrXc*, *params.thrNet11*, *params.thrNet12*, *params.thrNet22*. They are the same as those in function *'SNMNMF_comodule'*.

Output

W, H1, H2	Factorization results like those in function '*SNMNMF_comodule*'.
TerminalObj	A (*iter* $\times$ 3) matrix each row of which records the values of all the terms in the objective function and the sum of them in each iteration.

SNMNMF_module *Identify the md-modules from factorized matrix.*

Description

Based on the factorized matrix W, H_1 or H_2, identify the md-module members for each type of features.

Usage

module = SNMNMF_module(H, t, isdouble);

Arguments

H, t, isdouble	They are defined as those in function *'jNMF_module'*.

Output

module	Defined as that in function *'jNMF_module'*.

3.2 Output figures

SNMNMF_plot_X *Provide the heatmaps for the original input matrices.*

Description

Draw the heatmaps for the original input matrices (X_1 and X_2). The arguments and outputs of this function are similar with those in the function *'jNMF_plot_X'* of jNMF with $N = 2$.

Usage

SNMNMF_plot_X(X, XInd, fig, figure_title, colormap_type);

SNMNMF_plot_results *Show the heatmaps of a identified md-module.*

Description

Show the heatmaps of a selected identified md-module (circled in yellow lines). The arguments and outputs of this function are similar with those in the function *'jNMF_plot_result'* with $N = 2$.

Usage

SNMNMF_plot_results(X, XInd, FeatureType, fig, figure_title, colormap_type, vectorForRank);

SNMNMF_plot_correlation *Demonstrate the correlations between the original data and the reconstructed one.*

Description

Demonstrate the correlations between the original data X_i and the reconstructed one *newX*$_i$ = WH_i using boxplot ($i = 1, 2$). The arguments and outputs of this function are similar with those in the function *'jNMF_plot_correlation'* with $N = 2$.

Usage

corrMat = SNMNMF_plot_correlation(X, newX, XInd, newXInd, fig, figure_title);

SNMNMF_plot_distribution *Demonstrate the module size distributions.*

Description

This function provides the histograms for the size distributions of two types of features in the identified md-modules. The arguments and outputs of this function are similar with those in the function *'jNMF_plot_distribution'* with $N = 2$.

Usage

SNMNMF_plot_distribution(nSample, XInd, Comodule, FeatureType, fig, figure_title);

3.3 Output text files

Index2LabelForModuleContent *Output the identified md-modules into text files.*

Description

This function is the same as that described in jNMF.

OutputModule2TXT *Output the feature indexes of the identified md-modules.*

Description

This function is the same as that in jNMF.

4 sMBPLS

sMBPLS (**s**parse **M**ulti-**B**lock **P**artial **L**east **S**quare) extends the standard PLS method to discover associations between multiple input matrices ($X_1, X_2, ..., X_N; N \geq 1, X_i \in \mathbb{R}^{p \times n_i}$ and a response matrix ($Y \in \mathbb{R}^{p \times m}$) in a sparse manner. It identifies md-modules in which a subset of heterogeneous input features jointly explain a subset of the response variables. This problem is defined as,

$$\max_{w_i,q,b_i} \text{cov}(t,u) - \sum_{i=1}^{N} \lambda_i \|w_i\|_1 - \tau \|q\|_1$$

$$\text{with } t_i = X_i w_i, u = Yq, t = \sum_{i=1}^{N} b_i t_i$$

$$\text{s.t. } \|w_i\|_2^2 = 1, \|q\|_2^2 = 1, \sum_{i=1}^{N} b_i^2 = 1.$$

4.1 Algorithm

Run_sMBPLS	*The main function for sMBPLS.*

Description

This is the main function for sMBPLS which integrates all the related functions to achieve it.

Usage

Run_sMBPLS(Input);

Arguments

Input	A structure variable (Section 6).

Output

It saves all the results in the directory *'./MIA/sMBPLS/sMBPLS_Results/'*, including *sMBPLS_Results.mat, sMBPLS_RunRecords.txt, sMBPLS_Results.txt*, several folders and figures, which record the similar contents with those in function *'Run_jNMF'* of jNMF as shown in Figure 2, Figure 3 and Figure 4.

meanc	*Preprocess the input matrices.*

Description

This function centers the input data across the samples to enable the mean of each column of the input matrix to be zero.

Usage

[Zm,mz] = meanc(Z);

Arguments

Z	A matrix.

Output

Zm	The centered matrix.
mz	A row vector. *mz(i)* is the mean of *Z(:,i)*.

sMBPLS_comodule	*Obtain the md-modules.*

Description

This function returns all the md-modules by running SMBPLS for multiple times.

Usage

[nfactor, W, Q, WT, WU, TT, UU, sT, sU, XX, YY, Comodule, params] =
sMBPLS_comodule(X, Y, XInd, YInd, FeatureType, params);

Arguments

X	The input matrix of size ($p \times n$) combing N data blocks (e.g., $X = [X_1, ..., X_N]$).
Y	The response matrix of size ($p \times m$) combing M data blocks (e.g., $Y = [Y_1, ..., Y_M]$). Generally, $M = 1$.
XInd	A matrix of size $N \times 2$. The two elements of the ith row give the start and end column indexes in *X* for data matrix X_i $(i = 1, ..., N)$.
YInd	A matrix of size $M \times 2$. It is for Y defined similarly with *XInd*.
FeatureType	A ($1 \times (N + M)$) cell array. FeatureType{i} records the name of ith type of features (e.g., *FeatureType* = {*'microRNA expression','CNV', 'DNA Methylation','Gene expression'*}.
params	A structure variable including
params.nfactor	A pre-defined number of identified md-modules.
params.nfold	A positive integer which is the number of folds used for cross-validation (CV) procedures. Generally, we set *params.nfold* $= 5$ or 10.
params.maxiter	The maximal number of iterations for sMBPLS algorithm.
params.tol	The precision for the convergence of sMBPLS algorithm.
params.param	A ($l \times 1$) cell array restoring all the combinations of parameters to be selected. *params.param*$\{i\}$ contains one group of parameters used in the algorithm, including thrXc and thrYc (controlling the number of selected features of X and Y in the identified md-modules), thrXr and thrYr (thrXr = thrYr, controlling the number of selected samples in the identified md-modules), maxiter (maxiter = params.maxiter), tol (tol = params.tol).

Output

nfactor	The number of identified md-modules (*nfactor* $\leq$ *params.nfactor*).
W	A ($n\times$ *nfactor*) matrix, *W(:,i)* is the weight vector for X in ith md-module.
Q	A ($m\times$ *nfactor*) matrix, *Q(:,i)* is the weight vector for Y in ith md-module.
WT	A ($N\times$ *nfactor*) matrix, *WT(:,i)* is the super weight vector for X in ith md-module.
WU	A ($M\times$ *nfactor*) matrix, *WU(:,i)* is the super weight vector for Y in ith md-module.
TT	A ($p\times$ ($N\times$ *nfactor*)) matrix, $TT(:, N \times (i-1) + j)$ is the score vector for X_j in ith md-module ($i = 1, ...,$*nfactor*; $j = 1, ..., N$).
UU	A ($p\times$ ($M\times$ *nfactor*)) matrix, $UU(:, M \times (i-1) + j)$ is the score vector for Y_j in ith md-module ($i = 1, ...,$*nfactor*; $j = 1, ..., M$). Generally, $M = 1$.
sT	A ($p\times$ nfactor)) matrix, sT$(:, i)$ is the super score for X in ith md-module ($i = 1, ...,$nfactor).
sU	A ($p\times$ *nfactor*)) matrix, *sU*$(:, i)$ is the super score for Y in ith md-module ($i = 1, ...,$*nfactor*).

XX, YY	The new matrix after removing the signals of identified md-modules from X and Y.
Comodule	The md-modules are recorded in a (*nfactor*$\times(M+N+1)$) cell array which is similar to that in the function *'jNMF_comodule'*.
params	Compared to 'params' as input, there are something new added in it, including
params.iterNumList	A (*nfactor* $\times 1$) vector, where each element is the number of iterations for each round of running.
params.records	A (*nfactor* $\times 1$) cell array. *params.records*$\{i\}$ is a (*iter*$\times(M+N+2)$) matrix where each row records the values of all the terms in the objective function and the sum of them in each iteration when identify the ith md-module, and *iter = params.iterNumList(i), i = 1,...,nfactor*.
params.randRowPartitions	A (*params.nfold* $\times 1$) cell array. For n-fold CV procedure, p samples are partitioned into *params.nfold* groups equally. *params.randRowPartitions*$\{i\}$ records the sample indexes in the ith group ($i = 1, ...,$*params.nfold*).
params.cv_scores	A matrix where *params.cv_scores(*i, j*)* is the cv score for the ith group of parameters when identify the jth md-module.
params.paramsidx_used	A (*nfactor* $\times 1$) vector. *params.paramsidx_used(i)* is the index of selected parameters for identifying the ith md-module.

---

sMBPLS_algorithm *sMBPLS algorithm.*

Description

This implements the sMBPLS algorithm.

Usage

```
[success, w, q, b, a, T, U, t, u, XX, YY, TerminalObj, iter]
        = sMBPLS_algorithm(X, Y, XInd, YInd, param);
```

Arguments

X, Y, XInd, YInd	Defined the same as those in function *'sMBPLS_comodule'*.
param	A structure variable including six components:
param.maxiter, param.tol	Same as those in function *'sMBPLS_comodule'*.
param.thrXc, param.thrYc	Two positive integers which are two thresholds for selecting features of *X* and *Y* in the identified md-modules.
param.thrXr, param.thrYr	*param.thrXr = param.thrYr*. A positive integer which is a threshold for selecting samples in the identified md-modules.

Output

success	An indicator to show if this algorithm runs successfully or not (1 for success, 0 for failure).
w, q	Weight vectors for X, Y.
b, a	Super weight vectors for X, Y.
T	A ($p \times N$) matrix, *T(:,i)* is the score vector for X_i ($i = 1, ..., N$).
U	A ($p \times M$) matrix, *U(:,i)* is the score vector for Y_i ($i = 1, ..., M$). Generally, $M = 1$.

t, u	Super score vectors for X, Y.
XX, YY	The new matrices after removing the signals of current md-modules from X and Y.
iter	The number of iterations to indicate when the algorithm stops.
TerminalObj	A (*iter*$\times(2+N+M)$) matrix in which each row records the values of all the terms in the objective function and the sum of them in each iteration.

---

sMBPLS_params — *Integrate all the parameters into an unified framework.*

Description

This function is used to incorporate all the parameters into a structure variable *'newparams'*.

Usage

newparams = sMBPLS_params(params);

Arguments

params — A structure variable including

params.maxiter, params.tol, params.nfold, params.nfactor
: They are the same as those in the function *'sMBPLS_comodule'*.

params.thrXYr_list
: This is a column vector recording the thresholds for selection samples.

params.thrXc_list
: A $(1 \times N)$ cell array. *params.thrXc_list*$\{i\}$ is a column vector, which includes several thresholds for selection features in data matrix X_i $(i = 1, ..., N)$.

params.thrYc_list
: A $(1 \times M)$ cell array. *params.thrYc_list*$\{i\}$ is a column vector, which includes several thresholds for selection features in Y_i $(i = 1, ..., M)$.

Output

newparams — A structure variable which is the same as the input argument *'params'* in *'sMBPLS_comodule'*.

---

sMBPLS_select_param — *Select a group of proper parameters used in sMBPLS algorithm.*

Description

Using a cross-validation (CV) procedure to select a group of proper parameters.

Usage

[param_idx, cv_scores] = sMBPLS_select_param(X, Y, XInd, YInd, params);

Arguments

X, Y, XInd, YInd	Defined as those in function *'sMBPLS_comodule'*.
params	A structure variable including *params.param, params.randRowPartitions*, which are both the same as the output argument *'params'* in function *'sMBPLS_comodule'*.

Output

param_idx	The index of selected optimal parameters in params.param.
cv_scores	A column vector recording the CV scores for all groups of parameters.

---

sMBPLS_getCVscore	*Calculate the CV score for one group of parameters.*

Description

By using a n-fold cross-validation (CV) procedure for one group of parameters, it will obtain the corresponding CV score to assess this group of parameters. The smaller the better.

Usage

cv_score = sMBPLS_getCVscore(X, Y, XInd, YInd, param, randRowPartitions);

Arguments

X, Y, XInd, YInd, param	Defined the same as those in function *'sMBPLS_algorithm'*.
randRowPartitions	Defined the same as *'params.randRowPartitions'* in the output of function *'sMBPLS_comodule'*.

Output

cv_score	A score for this group of parameters *'param'* in the input arguments.

variable_sparse	*Make the vector sparse.*

Description

Make the input vector sparse based on the input threshold.

Usage

[sw, err] = variable_sparse(w, thrd, msg);

Arguments

w	A vector.
thrd	A threshold for the degree of sparsity of *w*.
msg	A string representing the name of variable *w*.

Output

sw	The sparse vector.
err	An indicator (1 indicates it is done successfully, and 0 for not).

newMatrix	*Obtain a new matrix.*

Description

Remove the signals of the current identified md-module from the current data matrix *X*, and obtain a new matrix *XX*.

Usage

XX = newMatrix(X, t, thrd, msg1, msg2);

Arguments

X	A matrix.
t	The latent variable for data *X*.
thrd	A threshold.
msg1	A string representing the name of variable *t*.
msg2	A string representing the name of variable *X*.

Output

XX	A new matrix removed signals from matrix *X*.

4.2 Output figures

---

sMBPLS_plot_XY	*Provide the heatmaps for the original or reordered input matrices.*

Description

Draw the heatmaps of the original input matrices (X, Y) by ignoring the input argument *'vectorForRank'* or the heatmaps of reordered input matrices ordered based on the variable *'vectorForRank'* where the signals of the identified md-module will be located in the four corners of this heatmap.

Usage

sMBPLS_plot_XY(X, Y, XInd, YInd, fig, figure_title, colormap_type, vectorForRank);

Arguments

X, Y, XInd, YInd — Defined as those in the function *'sMBPLS_comodule'*.

fig, figure_title, colormap_type
: Defined as those in the function *'jNMF_plot_X'* of jNMF.

vectorForRank — A structure variable including

- vectorForRank.t — The scores used for ordering samples. It is one column of matrix *sT* in the output of function *'sMBPLS_comodule'*.
- vectorForRank.w, vectorForRank.q
  : The weight vectors for X, Y related to a specific md-module, respectively. They are respectively one column of matrix *W, Q* in the output of function *'sMBPLS_comodule'*.

Output

The heatmaps for the original or reordered input data matrices.

---

sMBPLS_plot_results	*Demonstrate the heatmaps of a specific md-module and the scatterplots for the correlation between the selected features.*

Description

Show the heatmaps of a selected md-module (circled in yellow lines), and the scatterplots for the correlations between the selected features.

Usage

sMBPLS_plot_results(X, Y, XInd, YInd, FeatureType, fig, figure_title, colormap_type, vectorForRank);

Arguments

X, Y, XInd, YInd, fig, figure_title, colormap_type
: Defined the same as those input arguments in *'sMBPLS_plot_XY'*.

vectorForRank — A structure variable including six components. Except for the three ones in the input argument *vectorForRank* of *'sMBPLS_plot_XY'*, there are also

- vectorForRank.T — A $(p \times N)$ matrix. $T(:, i)$ is the score vector for X_i $(i = 1, ..., N)$.
- vectorForRank.U — A $(p \times M)$ matrix. $U(:, i)$ is the score vector for Y_i $(i = 1, ..., M)$.
- vectorForRank.comodule
  : A (1×$(N + M + 1)$) cell array. *vectorForRank.comodule*$\{i\}$ records the ith feature indexes of a selected md-module. The first column is for selected samples.

FeatureType — A (1×$(N + M)$) cell array. FeatureType{i} records the name of ith type of features (e.g., *FeatureType* = {*'microRNA expression','CNV',*

'DNA Methylation','Gene expression'}.

Output

The heatmaps for certain selected md-module to demonstrate the patterns of this md-module as shown in Figure 4.

The scatterplots for the correlation between the selected features as shown in Figure 5.

---

sMBPLS_plot_distribution *Demonstrate the module size distributions.*

Description

This function provides histograms for the size distributions of all the components in the identified md-modules.

Usage

sMBPLS_plot_distribution(nSample, Ind, Comodule, FeatureType, fig, figure_title);

Arguments

Ind *Ind = [XInd; YInd]. XInd, YInd* are the same as those in function *'sMBPLS_comodule'*.

Comodule It is the same as that in the output of function *'sMBPLS_comodule'*.

nSample, FeatureType, fig, figure_title
Similar with those in function *'jNMF_plot_distribution'*.

Output

Histograms for the size distributions of all the features in the identified md-modules.

4.3 Output text files

Index2LabelForModuleContent *Output the identified md-modules into text files.*

Description

This function is the same as that described in jNMF.

OutputModule2TXT *Output the feature indexes of the identified md-modules.*

Description

This function is the same as that described in jNMF.

5 SNPLS

SNPLS (**S**parse **N**etwork-regularized **PLS**) is designed for one input matrix ($X \in \mathbb{R}^{p\times n}$) and one response matrix ($Y \in \mathbb{R}^{p\times m}$). It introduces network-regularized constraints, expressed as adjacency matrices $A \in \mathbb{R}^{n\times n}$ of a given interaction network G_1 for the features in X and/or $B \in \mathbb{R}^{m\times m}$ of another interaction network G_2 for Y. This problem is defined as,

$$\max_{g,d} cov(Xg, Yd) - \lambda_1 g^T L_X g - \lambda_2 d^T L_Y d - \lambda_3 ||g||_1 - \lambda_4 ||d||_1$$
$$s.t. \quad g^T g = 1,\ d^T d = 1.$$

where $u = Xg, v = Yd, L_X, L_Y$ are the symmetric Laplacian matrices of network G_1, G_2, repsectively.

5.1 Algorithm

Run_SNPLS	*The main function for SNPLS.*

Description

This is the main function for SNPLS, which integrates all the related functions to achieve it.

Usage

Run_SNPLS(Input);

Arguments

Input	A structure variable (Section 6).

Output

It saves all the results in the directory *'./MIA/SNPLS/SNPLS_Results/'*, including *SNPLS_Results.mat, SNPLS_RunRecords.txt, SNPLS_Results.txt,* several folders and figures, which record the similar contents with those in function *'Run_jNMF'* of jNMF as shown in Figure 2, Figure 3 and Figure 4.

SNPLS_comodule	*Obtain the co-modules (md-modules).*

Description

This function returns all the co-module (md-modules) by running SNPLS for multiple times.

Usage

[nfactor, G, D, U, V, XX, YY, Comodule, params] = SNPLS_comodule(X, Y, A, B, FeatureType, params);

Arguments

X	The input matrix of size ($p \times n$).
Y	The response matrix of size ($p \times m$).
A	The adjacency matrix for the interactions between the features in X, where $A = (a_{ij}), a_{ij} = 1$ if features i and j are linked; $a_{ij} = 0$, otherwise.
B	The adjacency matrix defined similar with A for the features in Y.
FeatureType	A (1×2) cell array. FeatureType{i} records the name of ith type of features (e.g., *FeatureType* = {*'microRNA expression','Gene expression'*}.
params	A structure variable including the components below:
params.nfactor, params.nfold, params.maxiter, params.tol	Defined the same as those in the function *'sMBPLS_comodule'*.
params.param	A ($l \times 1$) cell array recording all the combinations of parameters to be selected. params.parami contains one group of parameters used in the algorithm, including *thrXc* and *thrYc* (controlling the number of selected features of *X* and *Y* in the identified md-modules) *thrXNet* and *thrYNet* (parameters for the network-regularized constraints in the objective function), *maxiter* (*maxiter* = *params.maxiter*), *tol* (*tol* = *params.tol*).
params.thrd_module	A positive vector of size 1×3 which stores the thresholds for selecting samples and features in X and Y.

Output

nfactor, XX, YY, Comodule

Similar with those in the function *'sMBPLS_comodule'* with

$N = M = 1$.

G — A ($n\times$ *nfactor*) matrix, *G(:,i)* is the weight vector for X in the ith md-module.

D — A ($m\times$ *nfactor*) matrix, *D(:,i)* is the weight vector for Y in the ith md-module.

U — A ($p\times$ *nfactor*)) matrix, *U(:,i)* is the super score for X in the ith md-module ($i = 1, ...,$*nfactor*).

V — A ($p\times$ *nfactor*)) matrix, *V(:,i)* is the super score for Y in the ith md-module ($i = 1, ...,$*nfactor*).

params — Compared to *'params'* as input, there are new components including *params.iterNumList*, *params.records*, *params.randRowPartitions*, *params.cv_scores*, *params.paramsidx_used* defined the same as the output argument in the function *'sMBPLS_comodule'*.

SNPLS_algorithm — *SNPLS algorithm.*

Description

This implements the SNPLS algorithm.

Usage

[success, g, d, u, v, XX, YY, TerminalObj, iter] = SNPLS_algorithm(X, Y, LX, LY, param);

Arguments

X, Y — The same as those in function *'SNPLS_comodule'*.

LX, LY — Laplacian matrices of size ($n \times n$) and ($m \times m$) about the interaction network for the features in data X and Y, respectively.

param — A structure variable including

params.tol, params.maxiter
: Defined the same as those in the function *'SNPLS_comodule'*.

param.thrXc, param.thrYc
: Two non-negative numbers which are respectively related to the parameters λ_3, λ_4 in the objective function.

param.thrXNet, param.thrYNet
: Two non-negative numbers. *param.thrXNet* is the parameter λ_1 in the objective function used in the network-regularized constraint about X. *param.thrYNet* is similar with *param.thrXNet* used in the netowrk about Y if available.

If *param.thrXNet = param.thrYNet = 0*, this algorithm reduces to sMBPLS for pairwise case.

If *param.thrXc = param.thrYc = param.thrXNet = param.thrYNet = 0*, this algorithm reduces to the standard PLS.

Output

success, TerminalObj, iter, XX, YY
: They are the same as those in the function *'sMBPLS_algorithm'*.

g, d — Weight vectors for X and Y.

u, v — Score vectors for X and Y.

SNPLS_params — *Integrate all the parameters into an unified framework.*

Description

This function is used to incorporate all the parameters into a structure variable *'newparams'*.

Usage

newparams = SNPLS_params(params);

Arguments

params — A structure variable including

params.tol, params.maxiter, params.nfold, params.nfactor, params.thrd_module — Defined the same as those in the function *'SNPLS_comodule'*.

params.thrXNet_list — This is a column vector recording the parameters for the network constraint about X in the objective function.

params.thrYNet_list — This is a column vector recording the parameters for the network constraint about Y in the objective function.

params.thrXc_list — A column vector, which includes several thresholds for selecting features in data matrix X.

params.thrYc_list — A column vector, which includes several thresholds for selecting features in data matrix Y.

Output

newparams — A structure variable defined the same as the input argument *'params'* in the function *'SNPLS_comodule'*.

SNPLS_select_param — *Select a group of proper parameters used in SNPLS.*

Description

Using a cross-validation (CV) procedure to select a group of proper parameters.

Usage

[param_idx, cv_scores] = SNPLS_select_param(X, Y, LX, LY, params);

Arguments

X, Y, LX, LY — Defined the same as those in the function *'SNPLS_algorithm'*.

params — A structure variable including *params.param, params.randRowPartitions*, which are both the same as the output argument *'params'* in the function *'SNPLS_comodule'*.

Output

param_idx — The index of the selected optimal parameters in params.param.

cv_scores — A column vector recording the CV scores for all groups of parameters.

SNPLS_getCVscore — *Calculate the CV score for a group of parameters.*

Description

By using a n-fold cross-validation (CV) procedure for one group of parameters, it will obtain the related CV score to assess these parameters. The smaller the better.

Usage

cv_score = SNPLS_getCVscore(X, Y, LX, LY, param, randRowPartitions);

Arguments

X, Y, LX, LY, param are the same with those in the function *'SNPLS_algorithm'*.

randRowPartitions is the same with *'params.randRowPartitions'* in the output of function *'SNPLS_comodule'*.

Output

cv_score — The score for this group of parameters *'param'* in the input arguments.

PLS	*The standard PLS algorithm.*

Description

This function is used to produce the initial vectors for SNPLS.

Usage

[success, g, d, u, v] = PLS (X, Y);

Arguments

X, Y	Two input matrices for the PLS algorithm.

Output

success	An indicator to indicate whether PLS performs successfully or not (1 for success and 0 for failure).
g, d	The weight vectors for input data X and Y.
u, v	The score vectors for input data X and Y.

thresholding	*Make the input vector sparse.*

Description

Make the input vector sparse based on the input thresholds.

Usage

sw = thresholding(w,thrd,msg);

Arguments

w	A vector.
thrd	A threshold for the degree of sparsity of *w*.
msg	A string for the name of variable *w*.

Output

sw	The sparse vector.

5.2 Output figures

SNPLS_plot_XY	*Provide the heatmaps for the original or reordered input matrices.*

Description

It is a similar function as 'sMBPLS_plot_XY' described in sMBPLS.

Usage

SNPLS_plot_XY(X, Y, fig, figure_title, colormap_type, vectorForRank);

Arguments

X, Y	The same as those in the function *'SNPLS_comodule'*.
fig, figure title, colormap_type	Defined the same as those in the function *'jNMF_plot_X'* of jNMF.
vectorForRank	A structure variable including
vectorForRank.u	The scores used for ordering samples. It is one column of matrix *U* in the output of function *'SNPLS_comodule'*.
vectorForRank.g, vectorForRank.d	The weight vectors for X and Y related to one identified md-module,

respectively. They are respectively one column of matrix *G, D* in the outputs of function *'SNPLS_comodule'*.

Output

The heatmaps for the original or reordered input data matrices.

SNPLS_plot_results — *Demonstrate the heatmaps of certain identified md-module and the scatterplots for the correlation between the selected features.*

Description

It is a similar function with *'sMBPLS_plot_results'* described in sMBPLS.

Usage

SNPLS_plot_results(X, Y, FeatureType, fig, figure_title, colormap_type, vectorForRank);

Arguments

X, Y, fig, figure_title, colormap_type
: The same as those of function *'SNPLS_plot_XY'*.

vectorForRank
: A structure variable containing

vectorForRank.g, vectorForRank.d, vectorForRank.u
: Defined the same as those in the function *'SNPLS_plot_XY'*.

vectorForRank.comodule
: A (1×3) cell. It has the similar meaning with that in the function *'sMBPLS_plot_results'*.

vectorForRank.v
: The score vector for data Y. It is one column of matrix *V* in the output of function *'SNPLS_comodule'*.

FeatureType
: Similar definition with that in the function *'sMBPLS_plot_results'* with $N = M = 1$.

Output

The heatmaps for certain selected md-module to demonstrate the patterns of this md-module. The scatterplots for the correlation between the selected features.

SNPLS_plot_distribution — *Demonstrate the module size distributions.*

Description

This function provides histograms for the size distributions of all the components in the identified md-modules, which has the similar input arguments and output results.

Usage

SNPLS_plot_distribution(nSample, Ind, Comodule, FeatureType, fig, figure_title);

5.3 Output text files

Index2LabelForModuleContent — *Output the identified md-modules into text files.*

Description

This function is the same as that described in jNMF.

OutputModule2TXT — *Output the feature indexes of identified md-modules.*

Description

This function is the same as that described in jNMF.

6 Input data

MIA implements all four methods using the same structure variable to describe the input data. This variable, named *Input*, includes the following components:

Input.data: A matrix storing all the multi-dimensional data sequentially (e.g., *Input.data* $=$ $[X_1, ..., X_N]$). Each row corresponds to the genomics features of a specific sample. Each type of genomic data is assigned its own set of columns.

Input.XBlockInd: A matrix of size $N \times 2$. The two elements in ith row give the start and end column indexes in *Input.data* for the ith matrix X_i $(i = 1, ..., N)$.

Input.YBlockInd: A matrix storing the response matrix Y for sMBPLS and SNPLS. Its format is similar to *Input.XBlockInd*.

Input.netAdj: The symmetric adjacency matrix of a given network used for SNMNMF and SNPLS, where the features have the same order as in *Input.data*. This network combines the interactions between and within the variables in multiple types of data matrices. For example, for SNMNMF method, *Input.netAdj* = $[A_{11}, A_{12}; A_{12}^T, A_{22}]$, where A_{11}, A_{22} are respectively the adjacency matrices for the interaction networks within the features in data matrices X_1, X_2; A_{12} is for the interaction network between the two types of features. The element of this matrix equals to 1 for linked features in the network, and 0 otherwise.

Input.SampleLabel: A vector recording the labels of samples.

Input.FeatureLabel: A vector recording the feature names in *Input.data*. The ith label corresponds to the ith feature in *Input.data*.

Input.FeatureType: A vector recording the feature types in *Input.data*. For example, *Input.FeatureType* = $\{$*'Gene expression', 'microRNA expression', 'DNA methylation'*$\}$.

Input.params: A structure variable, storing all the parameters used in MIA.

For these four methods, there are three common parameters, including

- *Input.params.NCluster*: A pre-defined number of md-modules. For example, we may set *Input.params.NCluster* $= 20$.
- *Input.params.maxiter*: The maximal iteration times in the algorithm. For example, we may set *Input.params.maxiter* $= 100$.
- *Input.params.tol*: The precision for convergence of the algorithm. For example, we may set *Input.params.tol* $= 10^{-6}$.

Besides, each method has its own specific parameters.

For jNMF, there are two specific parameters:

- *Input.params.nloop*: The number of repeating times to run this algorithm. To obtain a robust and optimal solution, this algorithm is run for multiple times repeatedly, and the solution with the minimal value of objective function is accepted. For example, we may set *Input.params.nloop* $= 50$.
- *Input.params.thrd_module*: A non-negative vector of size $1 \times (N + 1)$ to select features in md-modules. *Input.params.thrd_module*(i+1) is the threshold for selecting the ith type of features in *Input.data* ($i = 1, ..., N$). The first one is for selecting samples. The larger they are, the smaller number of features are selected. Users can set it based on the size of md-modules they prefer to identify. For example, we may set *Input.params.thrd_module = ones(1,N+1)*.

For SNMNMF, except for *Input.params.nloop* and *Input.params.thrd_module*, there are also:

- *Input.params.thrNet11, Input.params.thrNet12, Input.params.thrNet22*: The three non-negative numbers are set for the parameters respectively related to the network constraints about network A_{11}, A_{12}, A_{22} in the objective function, where A_{11}, A_{22} are respectively the adjacency matrices for the interaction networks within the features in data matrix X_1, X_2; A_{12} is for the interaction network between the two types of features. User can choose which networks they prefer to use in the framework by setting the corresponding parameters non-zero. For example, if *Input.params.thrNet11* $= 0$, the network A_{11} will not be used.
- *Input.params.thrXr, Input.params.thrXc*: The two non-negative numbers are set for W-related, and H_i-related constraints respectively in the objective function. It controls the degree of sparsity of matrix W, H_i. For example, we may set *Input.params.thrXr* $= 10$, *Input.params.thrXc* $= 10$.

For sMBPLS, there are:

- *Input.params.nfold*: A positive number used for n-fold cross-validation (CV) procedure. Generally, we set *Input.params.nfold* $= 5$ or $= 10$. This method applies CV procedure to select an appropriate group of parameters from all the combinations of these parameter lists described below.
- *Input.params.thrXYr_list*: A column vector with positive integers. They are candidates for thresholds in order to select samples in md-modules. For example, we may set *Input.params.thrXYr_list* = [20;30].
- *Input.params.thrXc_list, Input.params.thrYc_list*: Two row vectors of size $1 \times N, 1 \times M$ with positive integers to control the degree of sparsity for the weight variables of input data X, Y, respectively. For example, we may set
Input.params.thrXc_list $=$ repmat$(\{[20; 30]\}, 1, N)$,
Input.params.thrYc_list $=$ repmat$(\{[20; 30]\}, 1, M)$,
where $N =$ *size(Input.XBlockInd,1)*, $M =$ *size(Input.YBlockInd,1)*.

For SNPLS, there are:

- *Input.params.nfold*: It is the same as that in sMBPLS.
- *Input.params.thrXc_list, Input.params.thrYc_list*: They have the same meaning as those in sMBPLS for the situation of $N = 1$, $M = 1$. Thus, they are defined as column vectors. For example, we may set *Input.params.thrXc_list* $= [0.01; 0.03; 0.05]$, *Input.params.thrYc_list* $= [0.1; 0.3; 0.5]$.
- *Input.params.thrXNet_list, Input.params.thrYNet_list*: The two column vectors with non-negative values. They have the similar function with *Input.params.thrNet11* in SNMNMF. *Input.params.thrXNet_list, Input.params.thrYNet_list* are respectively for the networks within the features in input data X, and response data Y. For example, we may set *Input.params.thrXNet_list* $= [1; 5]$; *Input.params.thrYNet_list* $= [1; 5]$.
- *Input.params.thrd_module*: It is a non-negative matrix of size 3×2. The first column *Input.params.thrd_module*$(i, 1)$ is the threshold for selecting the ith feature in *Input.data* $(i = 1, 2, 3)$. And the second column *Input.params.thrd_module*$(i, 2)$ is a percentage in case of no features selected using the threshold. The first row is for selecting samples. The larger the thresholds are, the smaller number of features are selected. Users can set it based on the size of md-modules they prefer to identify. For example, we may set *Input.params.thrd_module* $= [1, 0.5; 1, 0.5; 1, 0.5]$.

In addition, for the components that are not used in certain methods (e.g., *Input.YBlockInd* in jNMF and SNMNMF and *Input.netAdj* in jNMF and sMBPLS), users can set them null or just ignore them.

With this data structure, MIA is able to partition *Input.data* into corresponding data matrices as input for each method automatically.

Here, we provide an example of constructed input data for SNMNMF method. Suppose that one wants to identify 50 microRNA-gene co-modules by integrating gene expression profiles ($X_1 \in \mathbb{R}^{385\times12456}$) and micro-RNA expression profiles ($X_2 \in \mathbb{R}^{385\times559}$) across the same set of samples, as well as the gene interaction network G_1, gene-microRNA interaction network G_2. The network G_1 can be expressed by the adjacency matrix $A_{11} = (a_{ij})_{12456\times12456}$, where $a_{ij} = 1$ if gene i and gene j is linked in the network G_1. Similarly, G_2 is expressed by the adjacency matrix $A_{12} \in \mathbb{R}^{12456\times559}$. If the microRNA interaction network is not available, the corresponding adjacency matrix A_{22} is defined as $A_{22} = \text{zeros}(559, 559)$.

Then, we could define the input data *Input* as below:

Input.data $= [X_1, X_2]$;
Input.XBlockInd $= [1, 12456; 12457, 13015]$;
Input.YBlockInd $= [\ \ ]$;
Input.netAdj = $[A_{11}, A_{12}; {A_{12}}^T, A_{22}]$;
Input.SampleLabel = {'TCGA-24-1105-01A';...;'TCGA-13-0793-01A'};
Input.FeatureLabel = {'SFRS8';...;'SCN3A';'hsa-mir-488';...;'hsa-mir-874'};
Input.FeatureType = {'Gene','miRNA'};
Input.params.NCluster $= 50$;
Input.params.maxiter $= 100$;
Input.params.tol $= 10^{-6}$;
Input.params.nloop $= 5$;
Input.params.thrd_module = [1,7,7];
Input.params.thrNet11 $= 10^{-4}$; *Input.params.thrNet12* $= 0.01$; *Input.params.thrNet22* $= 0$;
Input.params.thrXr $= 10$; *Input.params.thrXc* $= 10$;

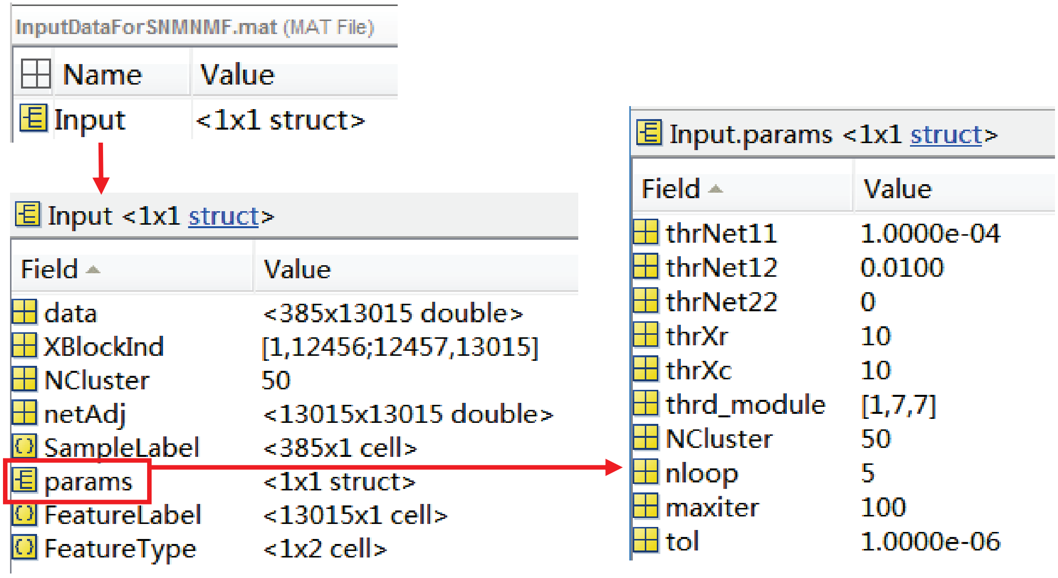

Figure 1: Illustration of an example of the input data for *SNMNMF*.

7 Output results

Given the input data and desired method, MIA automatically performs all computations and saves all the results in a specific folder named '****_Results*', where *** represents the pre-selected

method name. Here, we continue taking SNMNMF for example. We load the data constructed above and type these commands in the command window of MATLAB:

$>>$ *load('InputDataForSNMNMF.mat','Input')*

$>>$ *MIA(Input,'SNMNMF')*;

All the results are saved in the directory *'MIA/SNMNMF/SNMNMF_Results/'*. Referring to the results, there are four parts:

1) The first part is a MATLAB data file, named *'SNMNMF_Results.mat'*. In this file, we save the key variables calculated by this pre-selected method, such as solutions of the objective function, the parameters used in this method, the identified md-modules and so on (Figure 3c).

2) The second part contains some figures (Figure 2b, Figure 4 and Figure 5), including:

   - A heatmap of the input data named *'Original_data.fig'* that enables users to have basic understanding of input data;
   - An example for the heatmaps of identified md-modules (circled in yellow lines) and randomly selected features which show the patterns of the identified md-modules, saved as *'Identified_Comodule_*.fig'*, where * denotes the index of identified md-module (Figure 4 and Figure 5). The default value is 1. Users can change it by setting the value of variable *'SelectIDForExample'* in the function *'Run_jNMF'* or *'Run_SNMNMF'* to show certain md-modules. For sMBPLS and SNPLS, except for the heatmaps for co-modules, there are also scatter-plots to show the correlations between the latent variables related to input variables and response variables (Figure 5).
   - The size distribution for each type of components in identified md-modules, named *'Module_size_distributions.fig'* (Figure 4).
   - For jNMF and SNMNMF, we also provide the box-plots of sample-wise correlations of the original data and corresponding reconstructed data by factored matrices, named *'Correlations_between_original_space_and_reconstructed_space.fig'* (Figure 4). The large medians indicate that the dimension reduction technique captures the major information hidden in the original data.
   - For sMBPLS and SNPLS, we also provide heatmaps of reordered input data, named *'Reordered_data_*.fig'*, where * denotes the index of identified md-module (Figure 5). The reordered data are obtained by reordering the rows and columns of the original input data matrices such that the embedded module should be observed in left-top, left-bottom, right-top and right-bottom corners in the heatmaps.

3) The third part includes two text files (Figure 3d), *'SNMNMF_Results.txt'* and *'SNMNMF_RunRecords.txt'*. *'SNMNMF_Results.txt'* records the feature indexes of all the identified md-modules. It contains six columns, each of which is separated by comma. The first three columns are respectively the numbers of selected samples and two types of features, and the next three columns are the index lists which are included in the square brackets. *'SNMNMF_RunRecords.txt'* records the used parameters in the algorithm and the values of the objective function in each round of iterations.

4) The last part includes some folders, named as *'***Lists'*, where *** represents the feature type defined by input variable *'Input.FeatureType'* (e.g., 'Gene', 'microRNA'). In each folder, there are a number of text files, each of which is the list of one type of components in one identified md-module. For example, as shown in Figure 3a, it lists the selected gene names of the first identified module in *'GeneLists/GeneList_1.txt'*.

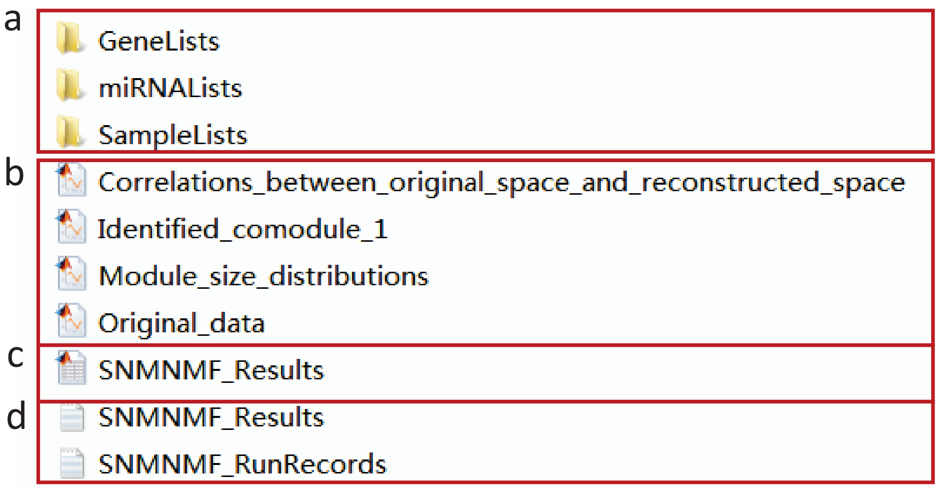

Figure 2: An overview of the output results for *SNMNMF*. The details about each part are shown in Figure 3 and Figure 4.

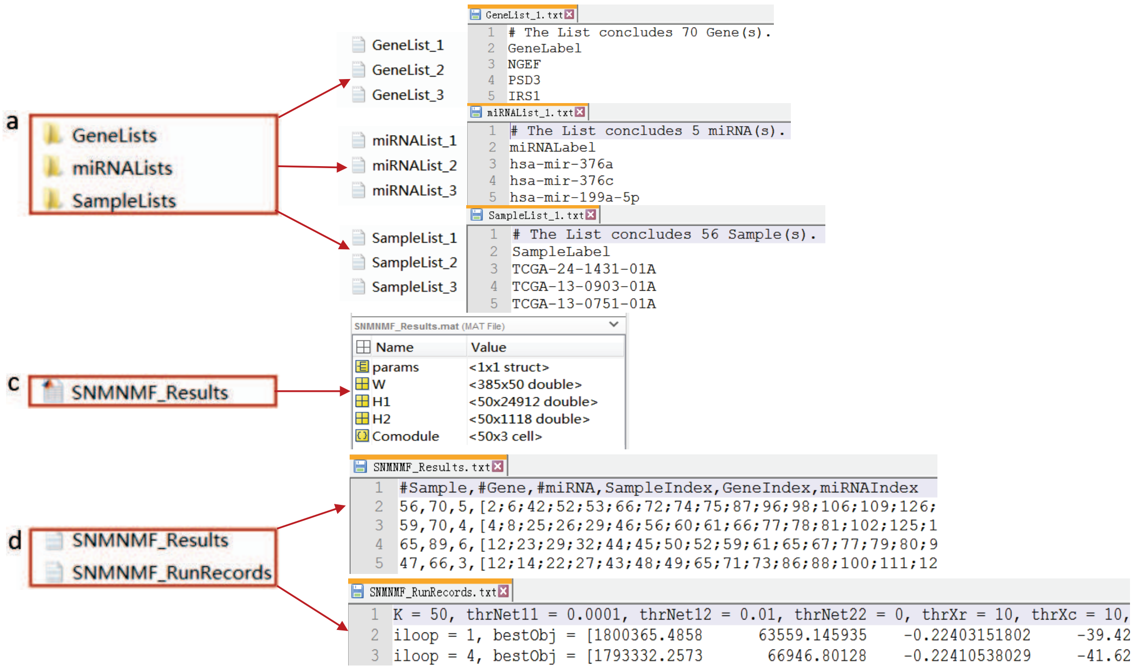

Figure 3: The details about the output files shown in Figure 2. (a) In each folder, there are a number of text files, each of which records one type of components in one identified co-modules. (c) A MATLAB data file storing the computation results, including the factored matrices $W, H1, H2$, the 50 identified co-modules, and the parameters used in this method. (d) The first text file records the feature indexes of all the identified md-modules, in which the first three columns are respectively the numbers of samples, genes, microRNAs in one identified md-modules, and the next three columns show the indexes of selected samples, genes and microRNA, which are included in the square brackets. The second text file records the used parameters in the algorithm (the first row) and the changes of the objective function during multiple-round running (the rest rows). It just keep track of the results if the objective value is smaller than that of the previous round. *bestObj* stores the value of each term in the objective function in the *'iloop'*th round, and *sum_Obj* is the sum of these terms.

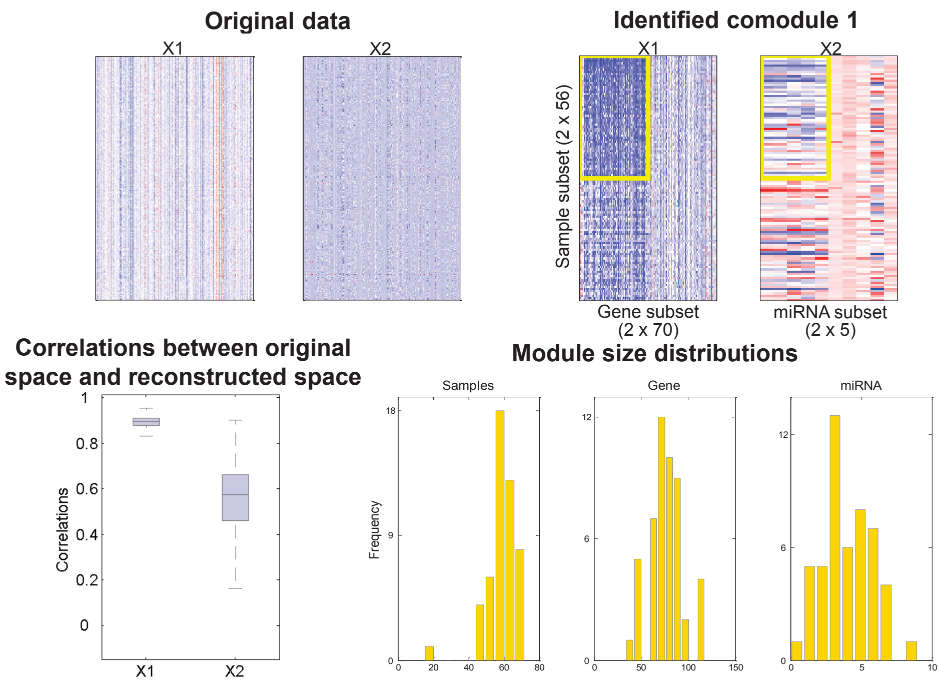

Figure 4: An example for the output figures in Figure 2b.

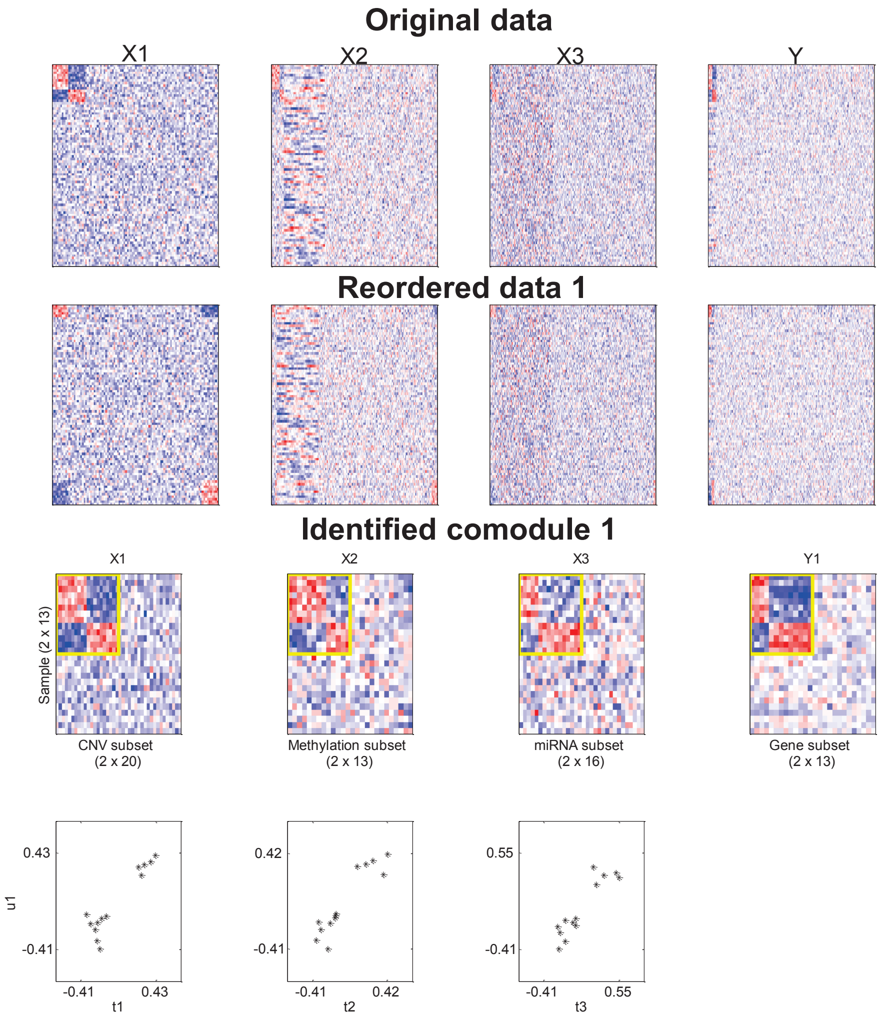

Figure 5: An example for the output figures for sMBPLS.
